# A long-term reconstruction of a global photosynthesis proxy over 1982–2023

**DOI:** 10.1038/s41597-025-04686-6

**Published:** 2025-03-03

**Authors:** Jianing Fang, Xu Lian, Youngryel Ryu, Sungchan Jeong, Chongya Jiang, Pierre Gentine

**Affiliations:** 1 Department of Earth and Environmental Engineering, Columbia University, New York, USA; 2 Interdisciplinary Program in Landscape Agriculture, Seoul National University, Seoul, Republic of Korea; 3 Integrated Major in Smart City Global Convergence, Seoul National University, Seoul, Republic of Korea; 4 Department of Landscape Architecture and Rural Systems Engineering, Seoul National University, Seoul, Republic of Korea; 5 Department of Natural Resources and Environmental Sciences, University of Illinois at Urbana-Champaign, Urbana, USA

**Keywords:** Carbon cycle, Climate-change ecology

## Abstract

Satellite-observed solar-induced chlorophyll fluorescence (SIF) is a powerful proxy for the photosynthetic characteristics of terrestrial ecosystems. Direct SIF observations are primarily limited to the recent decade, impeding their application in detecting long-term dynamics of ecosystem function. In this study, we leverage two surface reflectance bands available both from Advanced Very High-Resolution Radiometer (AVHRR, 1982–2023) and MODerate-resolution Imaging Spectroradiometer (MODIS, 2001–2023). Importantly, we calibrate and orbit-correct the AVHRR bands against their MODIS counterparts during their overlapping period. Using the long-term bias-corrected reflectance data from AVHRR and MODIS, a neural network is trained to produce a Long-term Continuous SIF-informed Photosynthesis Proxy (LCSPP) by emulating Orbiting Carbon Observatory-2 SIF, mapping it globally over the 1982–2023 period. Compared with previous SIF-informed photosynthesis proxies, LCSPP has similar skill but can be advantageously extended to the AVHRR period. Further comparison with three widely used vegetation indices (NDVI, kNDVI, NIRv) shows a higher or comparable correlation of LCSPP with satellite SIF and site-level GPP estimates across vegetation types, ensuring a greater capacity for representing long-term photosynthetic activity.

## Background & Summary

Photosynthesis by terrestrial vegetation is the largest driver of the interannual variability of global biogeochemical cycles, absorbing a considerable fraction of atmospheric CO_2_ annually and slowing down anthropogenic global warming^1^. Obtaining spatiotemporally continuous proxies of gross primary productivity (GPP) is crucial for understanding the climatic benefits of carbon cycle feedbacks, and for accurately forecasting forestry and agricultural yields^2^. Although the biochemistry of photosynthesis is well-characterized at the leaf level^3^, this carbon flux cannot be monitored directly via large-scale approaches such as spaceborne remote sensing. To date, major uncertainties still exist in the regional and global estimates of carbon uptake by terrestrial ecosystems^4^ and in the long-term retrieval of those carbon fluxes, limiting our understanding of the drivers of interannual and decadal variability in the biogeochemical cycle.

Traditionally, data-constrained global estimates of photosynthesis can be categorized into one of the following approaches: (1) empirical light-use efficiency models^5–7^; (2) simplified process-based radiative transfer and biochemical models^8^; (3) machine learning-based upscaling of site-level eddy-covariance flux measurements^9^; (4) data-assimilation approaches to constrain model posterior predictive fluxes with site-level and remote sensing observations^10^. Empirical light-use efficiency models use a light-use efficiency (LUE) assumption that approximates GPP as a product of the incident photosynthetically active radiation (PAR), the fraction of absorbed PAR (fPAR), and the LUE. These models use meteorological information to adjust the LUE and to generate GPP predictions at moderately fine temporal and spatial resolutions^7^. However, differences in how meteorological drivers are included in the formulations of LUE models result in disparate performance for simulating the temporal patterns of GPP on a regional scale or estimating the responses of GPP to drought events^11,12^. Process-based models can integrate the knowledge of the soil-vegetation-atmosphere continuum and resolve detailed process representations^3,13^, but the model’s complexity can make computations at high spatial and temporal resolutions prohibitively expensive^8^ and model tuning very challenging^14^. While developments in machine-learning models driven by Earth observations have vastly advanced our ability to upscale *in-situ* eddy-covariance carbon flux measurements globally^9^, systematic biases remain in simulating photosynthesis, especially in terms of interannual variabilities and the long-term trends of carbon uptake^15^. The biases of the data-driven machine-learning upscaling models are especially large in the wet tropics, limiting our ability to infer the trends and interannual variabilities of the global terrestrial carbon cycle^15^. Data-assimilation approaches adopt a Bayesian approach to constrain prior model parameters with observations, driven by the goal of deriving better predictions and principled uncertainty quantifications. Prominent examples of data assimilation models include but are not limited to ORCHIDAS^16^, CARDAMOM^17–20^, and CLM-DART^21^. Nevertheless, the quality of posterior predictive fluxes still depend on the information content of the observations assimilated^22^, the identifiability of the model parameters^18^, and potential structural uncertainties in the model formulation^23^.

The past decade has witnessed widespread application of satellite-observed passive solar-induced chlorophyll fluorescence as a proxy of gross primary productivity. Chlorophyll fluorescence is the emission of red and far-red photons from the excited states of Chl a molecules that provides an alternative energy dissipation pathway in addition to photochemical and nonphotochemical quenching^24,25^. Both leaf-level carbon assimilation (A_leaf_) and chlorophyll fluorescence (ChlF) can be written using LUE-based expressions^5^:

1 $${\rm{ChlF}}={\rm{PAR}}{\rm{\times }}{{\rm{fPAR}}}_{{\rm{chl}}}{\rm{\times }}{\Theta }_{{\rm{F}}}$$

2 $${{\rm{A}}}_{{\rm{leaf}}}={\rm{PAR}}{\rm{\times }}{{\rm{fPAR}}}_{{\rm{chl}}}{\rm{\times }}{\Theta }_{{\rm{P}},{\rm{leaf}}}$$

where Θ_F_ and Θ_P,leaf_ represent the quantum yield for ChlF emission and leaf-level photochemistry respectively. PAR stands for photosynthetically active radiation, while the fPAR_chl_ term represents the fraction of PAR absorbed by chlorophyll, which is a function of the canopy structure or the biophysical properties of vegetation. The product of the PAR and fPAR_chl_ terms constitutes PAR absorbed by the chlorophyll (APAR_chl_). When measured at ecosystem scale by spaceborne sensors, satellite SIF (SIF_sat_, Eq. 3) can be approximated with a similar equation with additional terms considering the fraction of SIF escaping from the canopy (f_esc_) and atmospheric transmittance (τ_atm_)^26^.

3 $${{\rm{SIF}}}_{{\rm{sat}}}={\rm{PAR}}{\rm{\times }}{{\rm{fPAR}}}_{{\rm{chl}}}{\rm{\times }}{\Theta }_{{\rm{F}}}{\rm{\times }}{{\rm{f}}}_{{\rm{esc}}}{\rm{\times }}{{\rm{\tau }}}_{{\rm{atm}}}$$

4 $${\rm{GPP}}={\rm{PAR}}{\rm{\times }}{{\rm{fPAR}}}_{{\rm{chl}}}{\rm{\times }}{\Theta }_{{\rm{P}},{\rm{canopy}}}$$

On a canopy to ecosystem scale, the rate of the total amount of organic carbon fixed by all green plants is captured by the notion of gross primary productivity (GPP). If we also take a light-use efficiency formulation of ecosystem productivity, then GPP can be expressed in the form of Eq. 4, where Θ_P, canopy_ is the canopy-scale photochemistry quantum yield (i.e., light use efficiency). Studies found that for crops and temperate deciduous forests, changes in APAR_chl_ and to a lesser extent f_esc_ primarily drive observed SIF variations, while Θ_F_ plays a minor role in the observed SIF dynamics across diurnal to seasonal scale^27–30^. In contrast, Θ_F_ variability has been shown more important in tracking the dynamics of GPP and photoprotective pigments in evergreen needleleaf forests^31,32^. Although the instantaneous SIF-GPP relationship can be non-linear — with GPP saturating at high solar irradiance and SIF largely scaling linearly with light^24,33,34^ — studies have found a robust near linear SIF-GPP relationship from daily to monthly scale using the OCO-2 SIF product^34,35^. These findings have encouraged the use of satellite-based SIF for many applications such as tracking dynamics of GPP^31^, phenological timing^36^ and crop yield^37^, and for carbon flux partitioning^38^, drought monitoring^39^, and assimilation into global biogeochemical models^19^.

Nevertheless, quantitatively connecting GPP to satellite SIF is hindered by the spatiotemporal discontinuities in the current generation of satellite SIF retrievals^25^, their low signal-to-noise ratio and their short record. The reliance on hyperspectral instruments for SIF entails a compromise between spectral resolution, sounding footprint size, and spatial continuity. For OCO-2, SIF is retrieved as individual soundings with a relatively fine footprint of 1.3 km × 2.25 km and large noise level^40^, but the wide gaps between neighboring swaths (~100 km) imply that the data often has to be aggregated to a coarse spatial resolution (about 1° × 1°) at a monthly scale^41^. While newer sensors such as TROPOMI provide near-continuous daily coverage of SIF observations thanks to a much wider swath and higher measurement frequency^42^, the wide range of solar zenith angle caused a large span of local solar time that can potentially complicate the signal interpretation^42^. Ongoing efforts to generate a multidecadal SIF record by harmonizing data from overlapping sensors demonstrate the potential for back-calibrating earlier SIF retrievals with more accurate TROPOMI and OCO-2 targets. However, further understanding of the uncertainties introduced by different retrieval methods and calibration drifts of GOME-2 sensors is needed to address the remaining inconsistencies^43^.

The spatiotemporal discontinuity, low spatial resolution and high noise of current satellite SIF products have motivated several studies to emulate the photosynthetic information embedded in SIF with broadband reflectance and complementary information of land cover and surface meteorology (see Table S1 in the SI for a summary of SIF-informed photosynthesis proxies). The overarching rationale for those efforts is that the main variations in satellite-based SIF originate from APAR_chl_, which can be approximated using satellite-observed optical reflectance channels^44–46^. Compared with empirical VIs (e.g., NDVI and MOD15 fAPAR) that mainly reflect total canopy fAPAR, SIF-informed photosynthesis proxies can better approximate fPAR_chl_ and are thus one step closer to tracking GPP^46^. As changes in fluorescence yield should mainly reflect short-term physiological signals, ideally Θ_F_ might be predicted with meteorological data. However, the effects of meteorological variables are insubstantial on the semi-monthly to monthly timescale as the SIF signal is mainly driven by slower-varying biochemical and canopy structural processes, making robust prediction of fluorescence yield challenging as Θ_F_ demonstrates minor variations under non-stressed conditions^30,46^. Furthermore, including meteorological data for predicting Θ_F_ may introduce circularity when linking SIF data with climate. A few studies found that including environmental variables as predictors did not significantly increase the prediction accuracy for SIF or Θ_F_^47^, which led the authors to construct models using reflectance data only^44,47,48^. We also elected not to include land cover type as model predictors because previous study showed minimal performance gain from using land cover datasets in SIF prediction when reflectance measurements are already included^49^. Nevertheless, we do caution that this emulation largely neglects the variations in Θ_F_, and the reconstructed photosynthesis proxies should not be conflated with measured SIF signal or used to derive fluorescence quantum yield.

The promise of SIF-informed photosynthesis proxies was confirmed by a recent intercomparison of four high-resolution SIF-informed photosynthesis proxies (CSIF, GOSIF, LUE-SIF, and HSIF) with GPP measurements from eddy-covariance methods. The study found that all SIF-informed photosynthesis proxies are unequivocally better predictors of site-level GPP than remotely sensed vegetation indices such as NDVI and EVI^50^. One remaining problem is the relatively short coverage of these SIF-informed photosynthesis proxies as they are trained with MODIS data, which are currently available for the last 20 years since the beginning of the MODIS era. Nevertheless, studies of phenological shifts, ecological droughts, and model calibration of biogeochemical processes would benefit from consistent, long-term, global observations of vegetation dynamics spanning multiple decades. In the absence of a long-term record of global SIF, researchers examining changes in ecological processes often turn to vegetation indices from earlier satellites to acquire a historical perspective of global change biology^51^.

Advanced Very-High-Resolution Radiometer (AVHRR) has been the most common suite of Earth sensors used for long-term vegetation studies before the MODIS era, given its global coverage, daily repeat cycles, and, most importantly, continuous availability since 1982. While the reliance on AVHRR for vegetation monitoring has been gradually superseded by newer satellites offering superior resolution, accuracy and consistency, the 40 years of AVHRR record still represents an invaluable dataset for studying decadal vegetation trends particularly before 2000. There is therefore substantial potential to build a long-term photosynthesis proxy by extending MODIS records to the 1980s using information from AVHRR. Nevertheless, some past studies have cautioned against a direct use of AVHRR data for long-term vegetation studies because it is plagued by several well-documented limitations, including spurious shifts in solar zenith angles (see Figure S1) due to orbital drifts^52^, inconsistencies between successive satellite sensors^52–54^, and the lack of on-board radiometric calibration^52^. These limitations necessitate carefully recalibrating the AVHRR reflectance.

In this study, we addressed the limitations in AVHRR reflectance by removing the orbital effects in AVHRR and cross-calibrating AVHRR against MODIS, in order to obtain a long-term consistent reflectance product. This new reflectance product was then used to define a Long-term Continuous SIF-informed Photosynthesis Proxy (LCSPP) from 1982–2023 at 0.05 degree spatial and biweekly temporal resolution. It is expected that the LCSPP can be used as a new dataset for evaluating long-term global vegetation dynamics under a changing climate.

In the first part of the manuscript, we present the methodology. In the second part, we examine the calibration of AVHRR dataset and compare LCSPP with several established vegetation productivity proxies. We then discuss how the surface reflectance and the reconstructed photosynthesis proxy can be used to study long-term vegetation dynamics, and highlight both the values and limitations of our product.

## Methods

**Fig. 1:**
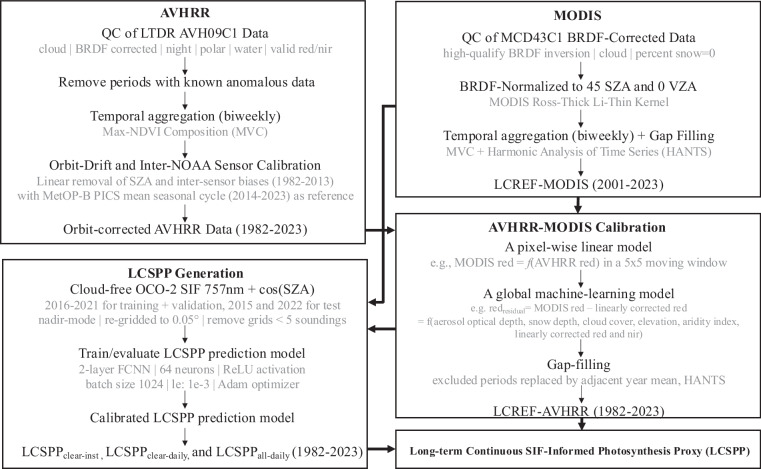
Flowchart showing the procedure of processing LCREF and the global reconstruction of LCSPP.

We calibrated and gap-filled AVHRR red and near-infrared reflectance channels from 1982–2023 to define a new temporally consistent Long-term Continuous REFlectance (LCREF-AVHRR) dataset at 0.05 degree spatial and biweekly temporal resolution. In addition, we also normalized, filtered, and gap-filled MODIS reflectance (LCREF-MODIS) to compare with concurrent AVHRR observations between 2001–2023. We trained a neural network for SIF emulation using only reflectance datasets and applied it separately on the AVHRR- and MODIS-based LCREF to globally map the Long-term Continuous SIF-informed Photosynthesis Proxy (LCSPP) from 1982–2023 (See Fig. 1 for the schematics). Finally, we evaluated LCSPP against existing photosynthesis proxies, VIs, and site-level GPP datasets.

### Datasets

#### Solar-induced chlorophyll fluorescence training data: OCO-2

The OCO-2 Solar Induced Chlorophyll Fluorescence Lite File V11r^55^ from 2015–2022 was used to train and test the machine learning model for the reconstruction of the LCSPP^41,56^. We followed the preprocessing procedure used in the production of CSIF to prepare the SIF data for the machine learning model^47^. The OCO-2 SIF retrievals were first filtered by measurement mode and the accompanying quality flags such that only the “best” quality clear-sky nadir mode measurements were used to develop the machine learning model. Next, the SIF data were aggregated to 0.05° daily grid cells to match the spatial resolution of the MODIS and AVHRR reflectance products^47^. Aggregation also reduces the random noise in the SIF retrievals by a factor of 1/sqrt(n), where n is the number of soundings in each grid cell. To limit the random uncertainties in the training targets, we further excluded grid cells with fewer than five soundings^47^. To examine whether the model performance was sensitive to the minimum OCO-2 soundings threshold, we also trained an alternative model using only grid cells with at least eight SIF retrievals. The SIF at 757 nm was selected for the reconstruction of LCSPP because it has a stronger correlation with GPP than the SIF at 771nm^57^. We used the SIF data from 2016 to 2021 for model training (n = 6,661,571 grid cells) and the data collected in the years 2015 and 2022 for testing (n = 2,247,733 grid cells). We selected test data before and after the training period to avoid potential performance overestimation caused by temporal autocorrelation in the datasets. In addition, we randomly subsampled 20% of the training data to create a validation dataset for hyperparameter tuning during model development. Figure S2 shows the spatial distribution of the training and test samples, and Figure S3 shows the distribution of OCO-2 soundings per grid cell in the training dataset.

#### LTDR AVHRR surface reflectance

Multiple land-surface reflectance and VI products have been developed from AVHRR GAC level-1b, including the Pathfinder AVHRR Land^58^, Global Inventory Modeling and Mapping Studies (GIMMS)^59^, the updated GIMMS3g dataset^60^, and the Land Long Term Data Record (LTDR)^61^, each with a different data-sensor lineage and calibration procedure. For this study, we selected the LTDR product because it provides daily gridded reflectance measurements at the same spatial resolution as MCD43C1, and our preliminary analysis shows it suffers less from orbital effects than the GIMMS series. We downloaded the daily 0.05° LTDR V5 AVH09C1 dataset^62–69^ between 1982–2021 from the NASA LAADS DAAC archive (https://ladsweb.modaps.eosdis.nasa.gov/missions-and-measurements/applications/ltdr/#project-documentation)^61^. The LTDR AVHRR surface product used the ocean and cloud vicarious radiometric calibration method to account for sensor degradations^70^, as well as the CLAVR-1 algorithm for cloud screening. Atmospheric corrections for molecular scattering, aerosol scattering, ozone absorption, and water-vapor absorption were applied in the data stream^71^. To minimize the signal variations caused by changing sun-sensor geometry, LTDR AVHRR was normalized to a standard observation geometry (solar zenith angle at 45°, view zenith angle at 0°, and relative azimuthal angle at 0°) using the well-established Vermote-Justice-Bréon (VJB) method and the Bidirectional Reflectance Distribution Function (BRDF) parameters derived from MODIS data^72–74^. We filtered the LTDR pixels using the QC flags for cloud, snow, BRDF correction, night, polar, water, and valid Red/NIR surface reflectance to retain only high-quality day-time observations with good BRDF correction. Details about the AVHRR sensor origin for each period are listed in Table S2.

#### MCD43C1 v061 MODIS/Terra + Aqua BRDF/Albedo Model Parameters

The MCD43C1 v061 MODIS/Terra + Aqua BRDF/Albedo Model Parameters^75^ were downloaded from LP DAAC. The MCD43C1 dataset was in a geographical coordinate grid with 0.05° spatial resolution based on a 16-day retrieval window. MCD43C1 contains weighting parameters used to drive the BRDF kernel for surface reflectance estimated from best-available observations over 16-day periods^76^. We computed surface reflectance at the standard observation geometry (45° SZA, 0° VZA, and 0° RAA) using the MODIS Ross-thick Li-sparse-reciprocal BRDF kernel implemented in the SASKTRAN 1.8.2 package^77^ to be consistent with the viewing geometry of the LTDR AVHRR dataset. We filtered the dataset to select cloud and snow-free pixels with high-quality BRDF-inversion flags (BRDF NBAR Quality ≤ 3).

#### Ancillary dataset

We used the following datasets to account for the potential covariates that may affect the cross-calibration between MODIS and AVHRR reflectance values. Temperature, precipitation, and incoming solar radiation dataset were extracted from the CRUNCEP data^78^. Cloud cover and snow depth samples were retrieved from the ERA5 reanalysis hourly dataset at 0.25°^79^. Aerosol optical depth (AOD) was obtained from the 3-hour 0.5 × 0.625° MERRA-2 reanalysis^80^ to control for aerosol effects, the Global 30 Arc-Second Elevation dataset was used to control for the topography (GTOPO30)^81^, and the 4 km Aridity Index (defined as potential precipitation divided by annual mean precipitation) computed from TerraClimate was used to control for surface aridity (as a measure for noises from background soils)^82^. All datasets were aggregated to bi-weekly temporal resolution and resampled to 0.05° using a cubic function.

### Preprocessing of AVHRR and MODIS reflectance to remove inter-sensor and orbital effects

We temporally aggregated the daily red and near-infrared bands of both MODIS and AVHRR to bi-weekly temporal resolution (1–15th for the first image, and 16th to the end of the month for the second image within the month) using the maximum value composite (MVC) based on NDVI^83^. The biweekly aggregation period was chosen as a balance between achieving sufficient temporal resolution to track seasonal to sub-seasonal vegetation dynamics and minimizing data gaps caused by frequent cloud cover and missing data in the AVHRR record. Three periods in the AVHRR record with either known instrumental malfunctions or anomalous observations were excluded from the calibration (See Figure S4 for the excluded periods, and SI Text 1 for a justification of anomalous data removal). As previous studies have confirmed MODIS to have good orbital stability and reliable radiometric calibration, we took it as ground truth to calibrate collocated AVHRR observations. While the LTDR surface reflectance product also applied the MODIS BRDF correction algorithm to rectify the spurious orbital drifts, past studies identified remaining orbital effects in the LTDR surface reflectance, which were propagated to down-stream NDVI, LAI, FPAR, and GPP products^84,85^. Furthermore, incomplete radiometric calibration results in systematic biases between individual AVHRR instruments^86^, particularly between AVHRR-2 and AVHRR-3 sensors (See Figure S4). We extracted LTDR reflectance averaged over 10 Pseudo-invariant Calibration Sites (PICSs) to quantify the inter-sensor biases. PICSs are non-vegetated desert targets selected based on their radiometric stability and high reflectance values to enable the calibration of space-borne sensors^87^. Here, we assumed that the reflectance anomalies at PICSs mostly originated from sensor biases^53,87^. Using the mean seasonal cycle of the most recent and radiometrically stable AVHRR-sensor onboard MetOP-B (MSC_MetOPB_PICS_red or nir_) as a reference, we computed the difference between the PICS-averaged reflectance series for all preceding AVHRR instruments with MSC_MetOPB_PICS_red or nir_ as a proxy of inter-sensor bias (Eq. 5)

5 $${\delta }_{{\rm{r}}{\rm{e}}{\rm{d}}{\rm{o}}{\rm{r}}{\rm{n}}{\rm{i}}{\rm{r}}}={{\rm{P}}{\rm{I}}{\rm{C}}{\rm{S}}}_{{\rm{r}}{\rm{e}}{\rm{d}}{\rm{o}}{\rm{r}}{\rm{n}}{\rm{i}}{\rm{r}}}{({\rm{N}}{\rm{O}}{\rm{A}}{\rm{A}})-{\rm{M}}{\rm{S}}{\rm{C}}{\rm{\_}}{\rm{M}}{\rm{e}}{\rm{t}}{\rm{O}}{\rm{P}}{\rm{B}}{\rm{\_}}{\rm{P}}{\rm{I}}{\rm{C}}{\rm{S}}}_{{\rm{r}}{\rm{e}}{\rm{d}}{\rm{o}}{\rm{r}}{\rm{n}}{\rm{i}}{\rm{r}}}$$

To remove the orbital effects and inter-sensor biases, we used an additional linear method to fit and then remove the contribution of solar zenith angle to the pixel-wise anomalies of surface reflectance in each of the red and near-infrared channels while retaining the explained anomalies by meteorological variables (Eqs. 6 and 7).

6 $${{\rm{\rho }}}_{{\rm{red\; or\; nir}}}={{\rm{a}}}_{1}{\rm{\times }}{\rm{SZA}}+{{\rm{a}}}_{2}{\rm{\times }}{{\rm{\delta }}}_{{\rm{red\; or\; nir}}}+{{\rm{a}}}_{3}{\rm{\times }}{\rm{Ta}}+{\rm{\times }}{{\rm{a}}}_{4}\times {\rm{P}}+{{\rm{a}}}_{5}{\rm{\times }}{\rm{Rad}}+{\rm{b}}$$

7 $${{\rm{\rho }}{\rm{\mbox{'}}}}_{{\rm{red\; or\; nir}}}={{\rm{\rho }}}_{{\rm{red\; or\; nir}}}-{{\rm{a}}}_{1}{\rm{\times }}{\rm{SZA}}-{{\rm{a}}}_{2}{\rm{\times }}{{\rm{\delta }}}_{{\rm{red\; or\; nir}}}$$

where ρ_red_ and ρ_nir_ are the anomalies in the LTDR Red and NIR channel, SZA is the anomaly of solar zenith angle at the time of acquisition, and T_a_, P, and Rad are air temperature at 2 m, total precipitation, and incoming solar radiation from the CRUNCEP dataset. We fitted the equation for each of the 24 biweekly periods within a year using ordinary least squares regression (OLSR) and used the regression coefficients of SZA and δ_red or nir_ to remove their contributions to reflectance for each pixel, obtaining the SZA and sensor-bias corrected reflectance (ρ’_red or nir_). These corrections were applied to AHVRR sensors onboard NOAA platforms between 1982–2013 only because the subsequent AVHRR sensor on MetOp-B (2014–2023) did not suffer from orbit drifts (Figure S4).

### Calibration of AVHRR reflectance bands against MODIS reflectance bands

We leveraged both the local and global correlational structures between overlapping MODIS and AVHRR observations over 2001–2023 to learn the (nonlinear) functional relationship between the two sensors and their respective channels. We first considered the local temporal correlation between the two instruments using a 5 × 5 running window to fit a pixel-wise linear model that maps AVHRR reflectance to MODIS values. The implementation of running windows was made to ensure enough sample size, particularly for humid ecosystems with frequency data gaps caused by heavy cloud cover. The pixel-wise linear models were fitted and applied separately to the AVHRR sensors onboard NOAA satellites (1982–2013) and MetOp-B (2014–2023). Next, we computed the residual between the MODIS value and this linearly-corrected AVHRR value (γ_residual_), which represents the remaining errors not captured by the locally adapted linear model. We assumed these differences could originate from the different non-linear responses of reflectance retrieved by MODIS and AVHRR instruments to environmental covariates such as atmospheric (aerosol and cloud cover) and surface conditions (topography, snow and soil background noises).

8 $${\gamma }_{{\rm{r}}{\rm{e}}{\rm{s}}{\rm{i}}{\rm{d}}{\rm{u}}{\rm{a}}{\rm{l}}}={\rm{M}}{\rm{O}}{\rm{D}}{\rm{I}}{\rm{S}}\,{\rm{v}}{\rm{a}}{\rm{l}}{\rm{u}}{\rm{e}}-{\rm{l}}{\rm{i}}{\rm{n}}{\rm{e}}{\rm{a}}{\rm{r}}{\rm{l}}{\rm{y}}\,{\rm{c}}{\rm{o}}{\rm{r}}{\rm{r}}{\rm{e}}{\rm{c}}{\rm{t}}{\rm{e}}{\rm{d}}\,{\rm{A}}{\rm{V}}{\rm{H}}{\rm{R}}{\rm{R}}\,{\rm{v}}{\rm{a}}{\rm{l}}{\rm{u}}{\rm{e}}$$

Building on this assumption, we trained neural networks (See SI Text 2 for details) at 0.05° resolution to capture the spatial relationship between γ_residual_ and environmental covariates including aerosol optical depth (AOD), snow depth (SD), cloud cover (CC), elevation (ELE), climatological aridity index (AI), and the linearly corrected AVHRR red and NIR reflectance. Geographical coordinates were not used as predictors to avoid overfitting due to spatial autocorrelations.

9 $${{\rm{\gamma }}}_{{\rm{residual}}}={\rm{f}}({\rm{AOD}},{\rm{SD}},{\rm{CC}},{\rm{ELE}},{\rm{AI}},{\rm{\text{linear corrected}}}\,{{\rm{\text{AVHRR}}}}_{{\rm{\text{red and NIR}}}})$$

We then applied both the pixel-wise linear model and the global-scale ML model for γ_residual_ to AVHRR observations prior to the MODIS era so that the double calibration could be carried out over to the entire time series. Note that double-calibration was conducted respectively for each of the 24 biweekly periods within a year (see Text S2 for technical details). After ML-correction, we filled each missing and excluded period with the seasonal mean of the preceding and following year. The remaining gaps in data were filled using the harmonic analysis of time series (HANTS) method, which applies a least squares curve fitting procedure for time series based on harmonic components of periodic functions^88^. We constructed two gap-filled reflectance products: LCREF-AVHRR using calibrated AVHRR reflectance from 1982–2023, and LCREF-MODIS using gap-filled and BRDF-normalized MODIS reflectance from 2001–2023. The two LCREF products were used for LCSPP reconstruction and the computation of various VIs for comparison in subsequent analyses.

### Reconstruction of LCSPP

We then trained feedforward neural networks to map the red and near-infrared bands from BRDF-normalized daily MODIS reflectance to the aggregated daily OCO-2 SIF observations. As SIF correlates strongly with incoming solar radiation, we used cos(SZA) as an additional predictor for the proxy of radiation, where SZA was the average of solar zenith angles for each OCO-2 SIF sounding in a grid cell. All predictors (inputs of the neural network) were standardized to accelerate the model convergence. We experimented with learning rates of 0.001 and 0.0005, number of hidden layers between 1–3, and hidden layer dimensions of 8, 32, and 64. We trained the model for 40 epochs. While we also tried regularization techniques such as dropout and early stopping, we found minimal advantages of those regularizations given the absence of apparent overfitting when evaluating the model on the validation data (see the OCO-2 section in Datasets description for how the data was split between training, validation, and test). The hyperparameter set that performed best on the validation dataset was then used to retrain the model with both training and validation data combined to maximize the number of samples. The retrained model was tested on both the held-out test dataset as a whole and on test data from different land cover types. Finally, we used this model to generate a biweekly global LCSPP_clear-inst_ product at 0.05° spatial resolution for 1982–2021 using the calibrated LCREF data and the instantaneous solar zenith angle based on the predicted OCO-2 satellite overpass time (with a nominal equator crossing time set 1:36 pm) for different dates and latitudes. This product provides an estimate of the instantaneous clear-sky solar-induced fluorescence at various locations, comparable with the SIF_clear-inst_ variable in the original CSIF product^47^. In addition, we also produced a LCSPP_clear-daily_ product based on the mean solar zenith angle over each day as a proxy of daily mean clear-sky solar-induced fluorescence consistent with the SIF_clear-daily_ variable in CSIF. The relation between LCSPP_clear-daily_ and LCSPP_clear-inst_ is based on a simple solar zenith angle correction with

10 $${LCSPP}_{clear-daily}={LCSPP}_{clear-inst}\times \frac{{\cos (SZA)}_{daily}}{{\cos (SZA)}_{inst}}={LCSPP}_{clear-inst}\times \frac{\frac{1}{24h}{\int }_{t={t}_{0}-12h}^{t={t}_{0}+12h}max(\cos (SZA(t)),0)dt}{\cos (SZA({t}_{0}))}$$

in which SZA(t_0_) is the expected instantaneous SZA at the time of OCO-2 overpass. Furthermore, we produced a third product of daily-averaged LCSPP adjusted by ERA5 surface solar radiation downward (i.e., including cloud cover effects), defined as

11 $${{LCSPP}}_{{all}-{daily}}=\frac{{{LCSPP}}_{{clear}-{inst}}}{{{PAR}}_{{clear}-{inst}}}\times {{PAR}}_{{daily}}^{{ERA}5}$$

where ${{PAR}}_{{clear} \mbox{-} {inst}}$ is an estimate of the clear-sky top-of-canopy radiation considering atmospheric scattering at the time of OCO-2 overpass (based on Appendix A1 in^47^), and ${{PAR}}_{{daily}}^{{ERA}5}$ is daily averaged surface solar radiation downward over each aggregation period of the reflectance dataset.

## Data Records

The main data output of this study is a Long-term Continuous SIF-informed Photosynthesis Proxy generated from the calibrated AVHRR record (LCSPP-AVHRR) from 1982–2023. We provide reconstruction of instantaneous clear-sky LCSPP (${LCSPP}_{clear \mbox{-} inst}$), daily mean clear-sky SIF (${LCSPP}_{clear \mbox{-} daily}$), and daily-mean all-sky SIF (${LCSPP}_{all \mbox{-} daily}$) as three proxies of vegetation activity. The latest version of LCSPP (v3.2) can be accessed at 10.5281/zenodo.7916850 for 1982–2000^89^, and 10.5281/zenodo.11906675 for 2001–2023^90^. In addition, we also provide the calibrated AVHRR red and NIR reflectance used to generate LCSPP-AVHRR as long-term continuous reflectance record (LCREF-AVHRR)^91^, publicly available at 10.5281/zenodo.11905959. Prospective users can readily compute common red and NIR based vegetation indices such NDVI, kNDVI, and NIRv suitable for their applications using LCREF-AVHRR.

We also made available MODIS-based LCSPP and LCREF to facilitate comparison with AVHRR for the overlapping period from 2001–2023, with the two LCSPP product generated using the same model. The file structures of LCSPP-MODIS^92^ (10.5281/zenodo.11658088) and LCREF-MODIS (10.5281/zenodo.11657458)^93^ are identical to the AVHRR versions. The reflectance datasets are normalized to the same standard sun-object-sensor geometry as the AVHRR product and gap-filled using the same approach.

All dataset outputs from this study are available at 0.05° spatial resolution and biweekly temporal resolution in NetCDF format. Each month is divided into two files, with the first file “a” representative of the 1^st^ day to the 15^th^ day of a month, and the second file “b” representative of the 16^th^ day to the last day of a month.

## Technical Validation

### Validation approach

We validated the orbital effects removal in the calibrated AVHRR reflectance record and the downstream LCSPP using 10 validation Pseudo-Invariant Calibration Sites (PICSs) in the Sahara Desert and on the Arabian Peninsula (see Table S3 for details). The validation PICSs differed from the 10 PICSs used for inter-sensor calibration for an independent assessment. To understand how each calibration step affected the AVHRR reflectance record, we extracted reflectance values from the original, intermediate, and calibrated AVHRR variables. MODIS reflectance was also evaluated against the PICSs to confirm its temporal consistency. We also investigated whether there remains to be systematic discrepancies between the successive sensors after calibration. In addition, we examined whether PICS-level reflectance anomalies in the LCREF datasets were correlated with the global interannual variabilities in LCSPP because any spurious trends introduced by orbital drifts, if present, might be manifested at a global scale.

Next, we investigated the prediction accuracy of our machine learning model for mapping from reflectance to SIF values on the held-out test dataset. To investigate whether the model has robust predictive power for samples from different vegetation types, we divided the test dataset into different vegetation types based on the majority International Geosphere-Biosphere Programme (IGBP) land cover type in the MCD12C1 dataset^94^ from 2015–2022. To ensure that a sufficient number of samples are available for each vegetation type, we combined evergreen needleleaf forest (ENF) and deciduous needleleaf forest into a single “needleleaf forest” (NF) type, aggregated closed shrubland and open shrubland into a single “shrubland” (SH) type, and merged woody savanna (WSV) and savanna (SAV) into a combined “savanna” (SAV) type. We used the root-mean-square error (RMSE) and the Nash-Sutchliffe Efficiency (NSE) between predicted values and observed SIF as metrics for model performance.

We then evaluated the R^2^ between LCSPP and site-level GPP measurements. We used the nighttime partition GPP estimate (GPP_NT_VUT_REF) at 165 eddy-covariance sites (See Table S4 for details) in the daily FLUXNET 2015 Tier One^95^ dataset as the benchmark, and we computed the R^2^ between site-level GPP and the VIs/photosynthesis proxies at the overlapping pixel after aggregation into biweekly temporal resolution to match with the LCREF and LCSPP datasets. We filtered all eddy-covariance data by the NEE QC flag such that a biweekly period was included in the analysis only if it contains at least 70% of measured or good quality gap-fill data (i.e., mean NEE_VUT_REF_QC > 0.7). To examine whether LCSPP could serve as a more robust proxy for GPP than other red and near-infrared bands-based VIs—NDVI^96^, kNDVI^97^, and NIRv^2^—we computed the R^2^ between VIs (calculated from LCREF) and site-level GPP as a comparison (see Text S3 for how we estimated the σ parameter in kNDVI)^98^. To account for the effects of sunlight variations on vegetation productivity not captured by reflectance measurements, we also compared LCSPP with NIRvP (computed as the product of NIRv and ERA-5 downward solar radiation). This recently developed proxy has been shown to robustly capture the spatiotemporal variations of SIF^27^.

Additionally, we examined the relationship between LCSPP and FLUXCOM, a machine-learning upscaling of site-level GPP. We utilized the state-of-the-art FLUXCOM-X-BASE^99,100^ product (2001–2014), aggregating hourly estimates to a biweekly time interval at the nearest 0.05° grid cell for each eddy-covariance tower. For pre-MODIS-era comparisons, we employed the earlier ANN-based RS + METEO FLUXCOM product^9,15^, which leveraged the climatology of various MODIS-derived datasets to capture spatial and seasonal variations, and CRUNCEPv6 data for temporal variability in meteorological forcings, as FLUXCOM-X-BASE was not available before 2000.

Our investigations encompassed both the original LTDR AVHRR dataset and LCREF-AVHRR to determine whether the calibration process enhances the correlations between satellite-derived and ground-based productivity estimates. We also conducted a comparative analysis of the correlation strength between LCREF-AVHRR-based variables and GPP before and after the year 2001. This allowed us to assess whether the benefits of the calibration procedure extended to the pre-MODIS era, utilizing a subset of 17 FLUXNET sites with at least three years of observations before and after the introduction of MODIS. All datasets were converted to a biweekly temporal resolution.

### Removal of AVHRR orbital effects

**Fig. 2:**
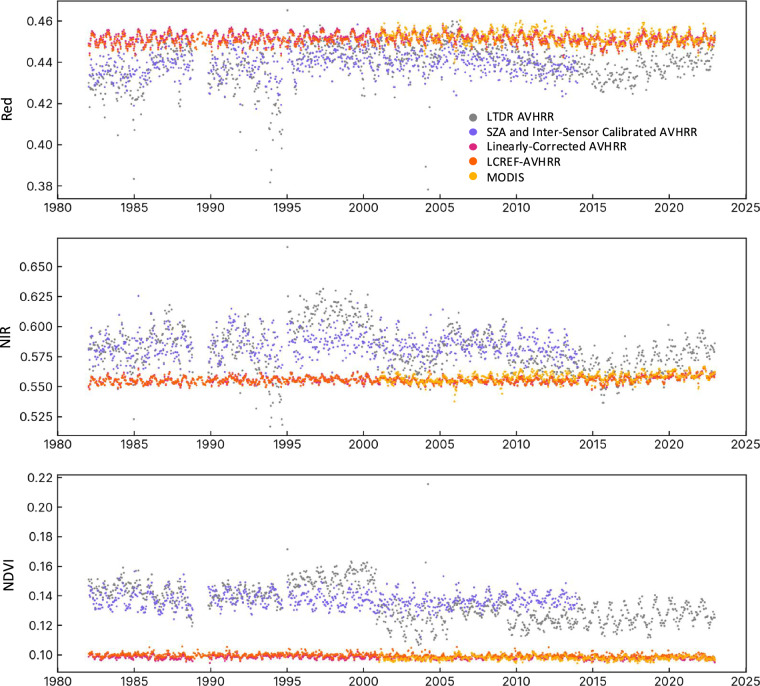
Comparison of MODIS and AVHRR reflectance against Pseudo-Invariant Calibration Sites (PICSs). In this figure, we present three panels depicting the median reflectance values observed at 10 validation PICSs for the red channel (top), the NIR channel (middle), and NDVI (bottom). LCREF-AVHRR is contrasted with the original LTDR AVHRR, as well as intermediate products obtained after SZA + inter-sensor calibration and pixelwise linear-calibration with MODIS. Additionally, we include the median reflectance and NDVI series from MODIS at the PICSs for comparison.

Our analysis begins with examining the removal of AVHRR orbital effects at the 10 PICSs reserved for validation. As Fig. 2 shows, the LTDR AVHRR reflectance over the calibration targets (shown in gray) exhibited large excursions punctuated by systematic discrepancies between AVHRR-2 and AVHRR-3 sensors, whereas the reflectance time series from the MODIS sensors (light orange) was much more stable. The orbital effects and inter-sensor bias correction (purple) notably enhanced the internal consistency within the AVHRR record. Moreover, implementing the pixel-wise linear and global machine-learning calibration with MODIS aligned the AVHRR record with its MODIS counterpart. Comparison of surface reflectance over the 12-month periods before and after each successive sensor change revealed that discrepancies due to sensor changes were reduced from as high as 5.59 ± 1.26% and 7.72 ± 1.28% (for N11 to N14, red and NIR site mean ± standard deviation) to less than 0.5% in all cases, aligning with the temporal stability observed between successive years at PICSs, as estimated using the benchmark MODIS record (Figure S5).

Our PICS analysis on the calibrated LCREF-AVHRR (Figure S6) also revealed that the magnitude of the residual orbital effects was small. The average annual detrended normalized anomalies for the calibrated red and near-infrared bands of the AVHRR reflectance were well within 1%. Furthermore, there were no significant correlations between the detrended normalized anomalies of reflectance values at the validation PICSs and the global anomalies of LCSPP. Specifically, the correlations for red reflectance vs. LCSPP (r = 0.191, *p* = 0.294) and NIR reflectance vs. LCSPP (r = 0.104, *p* = 0.571) were found to be non-significant (as shown in Figure S6).

To examine whether the orbital effects removal was successfully applied at different locations, we also analyzed the pixel-wise correlations between the detrended anomalies of annual mean SZA and that of corrected reflectance bands and LCSPP. Results indicated that although the SZA excursions due to orbital drifts were more pronounced in the tropics than in high-latitude regions (Figure S6d), our calibration procedure removed spurious correlations due to SZA shifts in a spatially consistent manner. This was evident as the remaining correlations with SZA for LCREF and LCSPP were weak in most regions (Figure S7a–c).

### SIF Emulation model performance

**Fig. 3:**
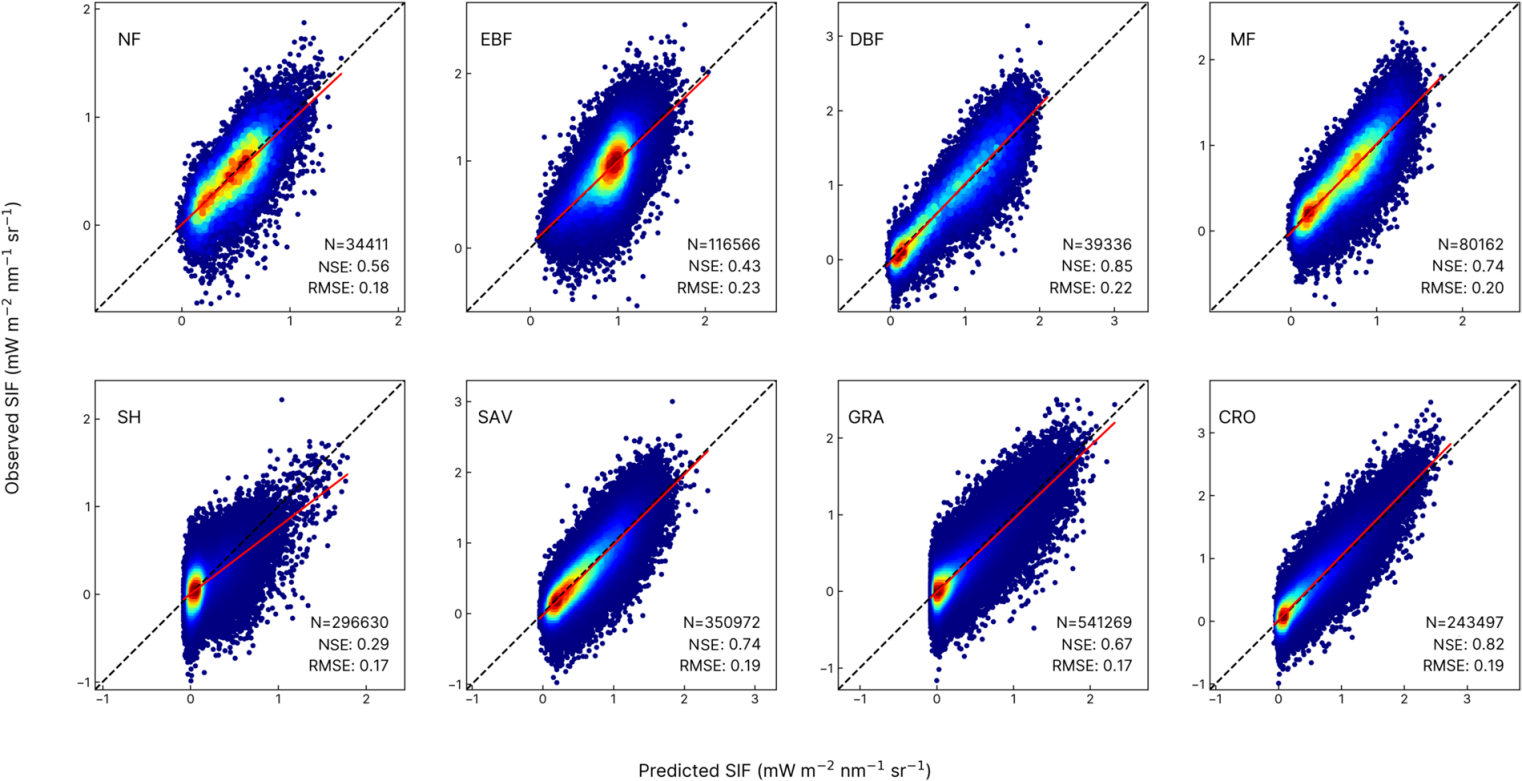
Evaluating the emulation of OCO-2 SIF on the test dataset by IGBP land classification types. NF: Needleleaf Forest (evergreen and deciduous needleleaf forest); EBF: Evergreen Broadleaf Forest; DBF: Deciduous Broadleaf Forest; MF: Mixed Forest; SH: Shrubland (open and closed shrubland); SAV: Savanna (savanna and woody savanna); GRA: Grassland; CRO: Cropland (See Figure S9 for results on the training dataset). Each dot represents a daily grid cell of a particular land classification type, and the regression includes all grid cells of one specific land classification type over the years.

The machine learning model used for SIF emulation attained good accuracy for statistically reproducing OCO-2 SIF, reaching a NSE of 0.79 and RMSE of 0.19 mW m^−2^ nm^−1^ sr^−1^ on the training dataset. The model also successfully captured the variations of OCO-2 SIF on the test dataset with an NSE of 0.79 and RMSE of 0.19 mW m^−2^ nm^−1^ sr^−1^, suggesting that the model showed minimal indication of overfitting (Figure S8). It is important to note that the predictive performance of our two-band model is comparable to that of the four-band algorithm used to produce CSIF (NSE = 0.796 and RMSE = 0.182 on the training dataset, NSE = 0.786 and RMSE = 0.177 on the test dataset) in terms of the correlation with OCO-2 soundings. We observed high correlations (NSE > 0.65) between the observed and model-predicted SIF (Fig. 3) for deciduous broadleaf forest (DBF), mixed forest (MF), savanna (SAV), grassland (GRA), and cropland (CRO). However, we noticed a lower correlation (NSE = 0.29) between predicted and observed SIF for the shrubland (SH), potentially due to the larger signal-to-noise ratios of SIF retrievals in sparely vegetated regions. In addition, we note slightly subpar correlations for needleleaf forest (NF, NSE = 0.56) and evergreen broadleaf forest (EBF, NSE = 0.43), probably caused by the weak seasonality in the canopy structure in these vegetation types (Fig. 3). Sensitivity tests showed the model performance was robust to selecting different years for model evaluation (Figure S10), and the model performance showed minimal improvement when it was trained on grid cells with at least 8 OCO-2 soundings compared with the current approach.

### Temporal and spatial pattern of LCSPP

**Fig. 4:**
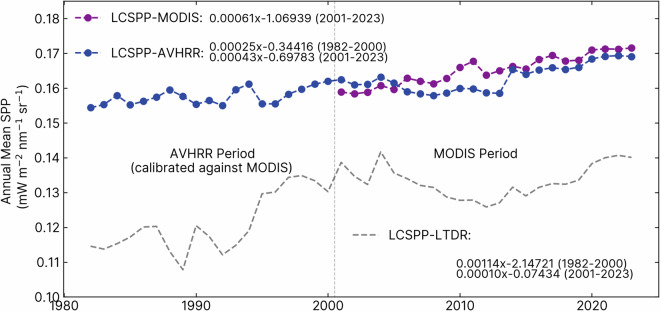
Annual mean growing season time series of AVHRR- and MODIS-based LCSPP_clear-daily_. LCSPP directly reconstructed from the original LTDR dataset is also included for comparison. Pixels with SZA < 0 (high-latitude winter) were assigned the value of 0 mW m^−2^ nm^−1^ sr^−1^. The equations in the plot are linear least-square regressions of annual mean LCSPP for each dataset. We first computed the mean monthly LCSPP for each pixel before aggregating it to annual mean values. A common growing season data mask was applied to all datasets, and the pixels were area-weighted during spatial aggregation. We define the growing season as months where the climatology mean temperature is above 5 °C.

We first conducted a comparative assessment of the mean temporal trends in calibrated AVHRR and MODIS-based LCSPP, as depicted in Fig. 4. LCSPP-AVHRR displayed a global increasing trend of 0.0025 mW m^−2^ nm^−1^ sr^−1^ per decade during the period from 1982 to 2000. Between 2001 and 2023, the trend in LCSPP-AVHRR appeared to dip slightly in the mid-2000s before resuming an upward trajectory in the 2010s, resulting in an overall increasing rate of 0.0043 mW m^−2^ nm^−1^ sr^−1^ per decade. In contrast, LCSPP-MODIS exhibited a more rapid upward trend, with a rate of 0.0061 mW m^−2^ nm^−1^ sr^−1^ per decade.

As a reference point for comparison, we examined LCSPP directly reconstructed using LTDR AVHRR data (LCSPP-LTDR). LCSPP-LTDR experienced a substantial increase between 1982 and 2000, with a rate of 0.0114 mW m^−2^ nm^−1^ sr^−1^ per decade, accompanied by notable fluctuations. However, the overall trend of SIF-LTDR was nearly flat from 2001 to 2023, driven by a significant decline in the 2000s, followed by a recovery in the subsequent decade. When compared with LCSPP-LTDR, LCSPP reconstructed using calibrated AVHRR reflectance exhibited considerably smaller interannual variabilities and more closely aligned with MODIS-derived values in both absolute value and trend.

To assess the impact of long-term solar radiation shifts^101^, we compared the normalized anomalies of clear-sky daily (LCSPP_clear-daily_) and all-sky daily SIF (LCSPP_all-daily_). Although the mean value of all-sky LCSPP is only ~75% of the clear-sky LCSPP value, their normalized anomalies are highly correlated (r > 0.98), suggesting that the trends in surface incoming solar radiation has only a minor impact on the long-term LCSPP trends.

**Fig. 5:**
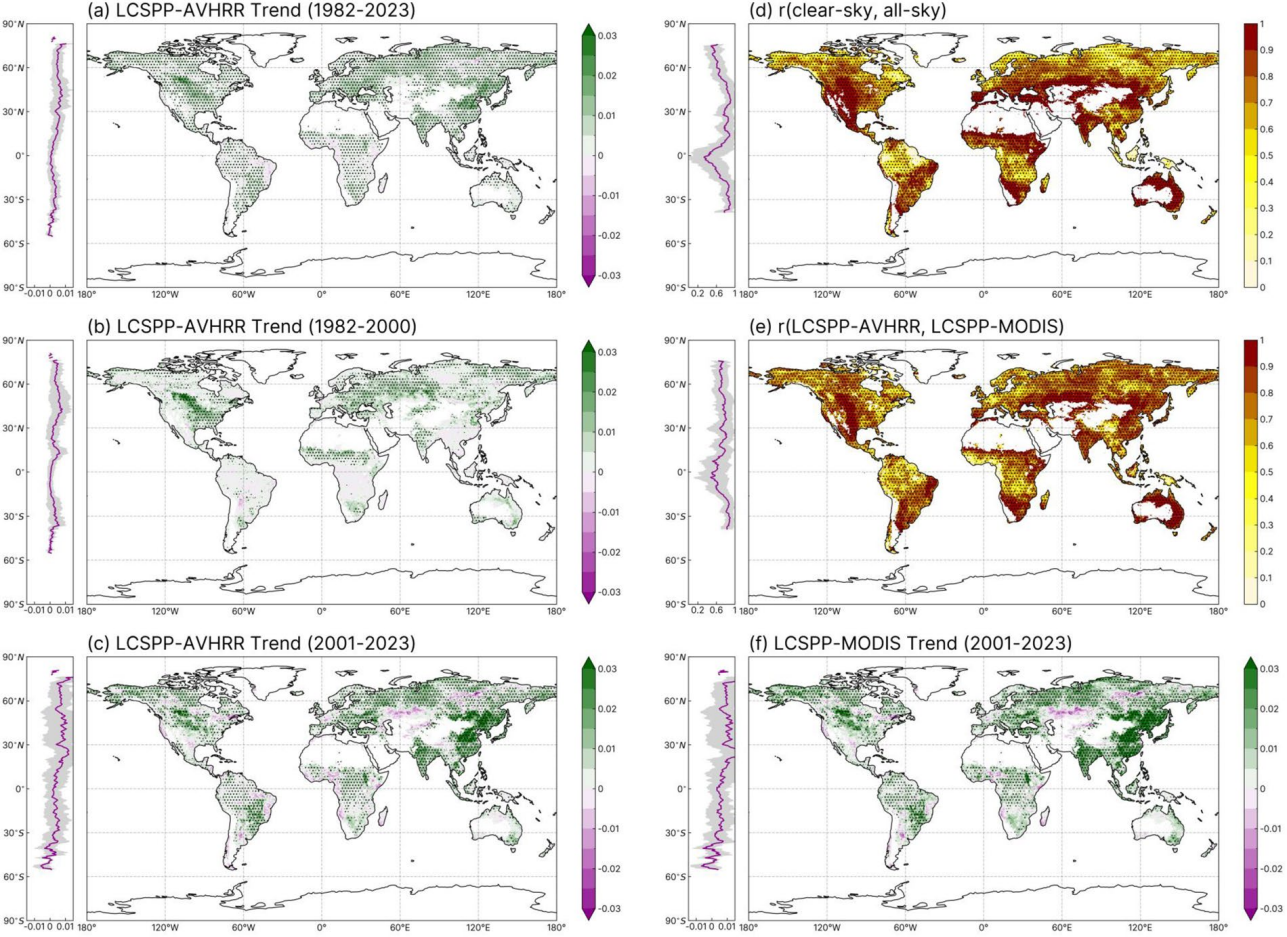
Spatial trends and correlations in AVHRR and MODIS-based LCSPP. (**a**) Depicts the spatial pattern of AVHRR-based LCSPP_clear-daily_ trends for the entire record spanning from 1982 to 2023, while panels (**b**) and (**c**) separate the trends for the periods 1982–2000 and 2001–2023, respectively. (**d**) Illustrates the annual-level correlation between clear-sky and all-sky daily LCSPP, and (**e**) provides the correlation between clear-sky LCSPP-AVHRR and LCSPP-MODIS. (**f**) Presents the MODIS-based LCSPP_clear-daily_ trends for the period 2001–2023. Each subplot includes an inset on the left, displaying the latitudinal distribution of trends or correlations along with their associated standard deviations. Areas with a multi-year average LCSPP < 0.03 mW m^−2^ sr^−1^ nm^−1^ have been excluded and are represented in white. Significance levels for both trends and correlations are determined through pixel-wise least square regression, with alpha set at 0.05. All trends and correlations are computed from annual growing season mean.

We next examined the spatial pattern of the LCSPP trends for both AVHRR and MODIS-based versions. During the 1982–2000 period, an increasing LCSPP-AVHRR trend was predominantly observed in eastern Europe, the Sahel, southern Africa, as well as in the Midwest and southeastern regions of the United States (Fig. 5b). While the trend of enhanced productivity in most of these regions continued into the early 21^st^ century, the most notable LCSPP increase during the 2001–2023 period was found in eastern China, India, and the soybean belt of South America (Fig. 5c). These regions with amplified LCSPP trends were the primary contributors to the accelerated global trend from 1982–2000 to 2001–2023 (Fig. 4). Furthermore, a rise in LCSPP was also evident across much of the boreal forest during the 2001–2023 period. By contrast, a slight browning pattern was noted in the eastern Amazon (Fig. 5c,f).

LCSPP-AVHRR and LCSPP-MODIS showed a generally consistent spatial trend during their overlapping period (Fig. 5c,f), despite the mild decline of LCSPP-AVHRR in mid-2000s, as observed in Fig. 4. The interannual variabilities of MODIS and AVHRR-based LCSPP were positively correlated over much of the global vegetated surface. However, the strength of the correlation appeared to be lower in the tropics and parts of the northern forest (Fig. 5e). Accounting for the cloud cover and aerosol effects on surface solar radiation led to a moderate reduction in the clear-sky vs. all-sky LCSPP-AVHRR correlations in the wet tropics and the high-latitude boreal region (Fig. 5d).

To ensure the robustness of the results, we also computed the pixel-wise LCSPP trend using the Theil-Sen slope estimator and tested the significance of the slope with the Hamed & Rao modified Mann-Kendall (MK) trend test, a robust non-parametric test to detect the monotonic upward and downward trend in serial data. The results from the MK test (Figure S11) were consistent with the spatial patterns in Fig. 5, confirming the statistical robustness of the derived trends.

### Comparison of LCSPP with site-level GPP

We evaluated the R^2^ between LCSPP and GPP estimated from eddy-covariance sites in the FLUXNET2015 datasets to investigate whether the LCSPP is a reliable proxy for GPP. As a benchmark for comparison, we computed the R^2^ between site-based GPP and satellite-based LCSPP and of three widely-adopted red and NIR band-based VIs: Normalized Difference Vegetation Index (NDVI)^96^, Near-infrared Reflectance of vegetation (NIRv)^2^, and kernel NDVI (kNDVI)^97^. Additionally, we also included a comparison of LCSPP with the recently-developed NIRvP^27^, which is computed as the product between NIRv and ERA-5 incoming solar radiation.

**Fig. 6:**
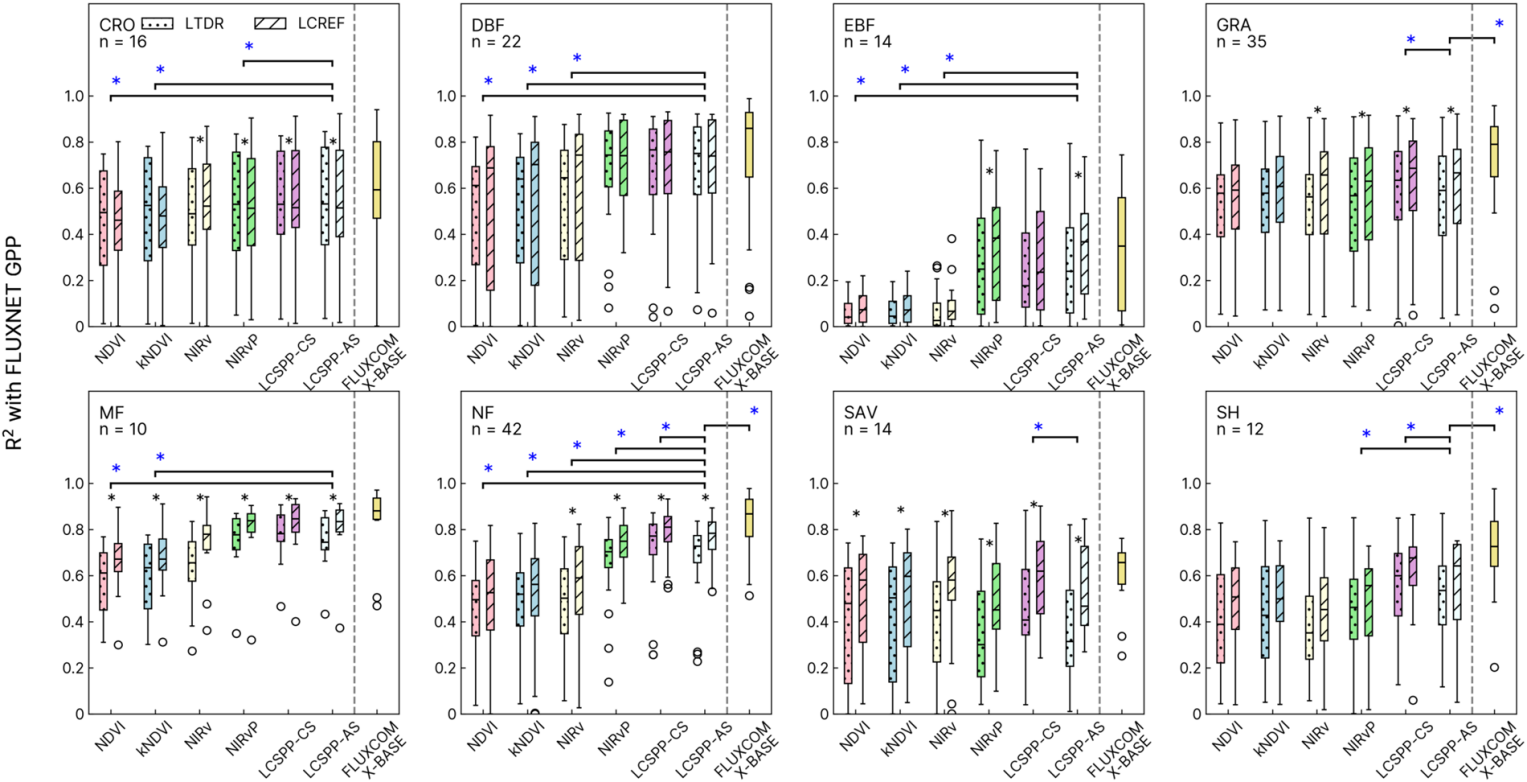
Comparative analysis of the calibrated AVHRR and the original LTDR AVHRR based VI-GPP and LCSPP-GPP correlations at eddy-covariance sites. For this analysis, we used nighttime partitioned GPP in the FLUXNET 2015 dataset starting from 2001. A blue asterisk signifies whether the correlation with GPP is stronger in the case of all-sky LCSPP (LCSPP-AS) compared to a reflectance-only VI, as determined by a paired t-test with a one-tailed *p*-value < 0.05. It also signifies whether there is a difference in the correlation with FLUXNET GPP between all-sky LCSPP (LCSPP-AS), FLUXCOM-X-BASE GPP, NIRvP, or clear-sky LCSPP (LCSPP-CS), as determined by a paired t-test with a two-tailed *p* < 0.05. The black asterisk indicates, for each type of VI or SIF-informed photosynthesis proxy, whether the calibrated LCREF-based version has a higher correlation with site-level GPP compared with the LTDR AVHRR-based product (one-tailed *p* < 0.05).

Our results demonstrated that LCSPP outperformed VIs that solely relied on reflectance information (NDVI, NIRv, kNDVI) across a wide range of vegetated land cover types, explaining 10–30% of additional variance in site-level GPP as measured by R^2^. However, the capability of LCSPP in capturing GPP variations was mostly on par with NIRvP, which benefits from the additional information about incoming solar radiation (Fig. 6). This similarity in performance confirms our expectation that the dominant signal in LCSPP derives from canopy structure and light variabilities, aligning with a property of SIF itself^27^. However, LCSPP demonstrates higher correlations with GPP at needleleaf forest sites than NIRvP, suggesting that training against OCO-2 SIF allows it to emulate at least some additional physiological signals in SIF that is not explained by canopy structure and incoming solar irradiance alone. LCSPP exhibited the greatest advantage over the VIs in the evergreen broadleaf forest (EBF) and needleleaf forest (NF), likely due to these vegetation classes having small seasonal canopy structure variations, which are the primary signals of VI variabilities. In addition, we did not observe a significant improvement in R^2^ after adjusting clear-sky daily SIF with all-sky incoming radiation, suggesting that cloud cover variations had only have a minor impact on a biweekly timescale.

A further comparison with FLUXCOM-X-BASE GPP suggested that LCSPP-AVHRR had generally comparable correlations with site-level GPP as the state-of-the-art FLUXCOM upscaling product at CRO, DBF, EBF, and MF sites, yet FLUXCOM-X-BASE has higher R^2^ at GRA, NF, and SH sites (*p* < 0.05, two-tailed paired-t test). As LCREF is mainly capturing APAR_chl_ variations but not fast changes of light-use efficiency, it misses short-term variabilities in the GPP dynamics compared to FLUXCOM-X-BASE, which has been directly trained against site-level GPP using both MODIS-based variables and hourly meteorological observations. However, we do note that the R^2^ in FLUXCOM-X-BASE might be partially inflated, as FLUXCOM X-BASE was trained on the same set of FLUXNET2015 sites used to evaluate the correlation with GPP in this analysis. It remains to be tested how SIF-informed photosynthesis proxies compare with upscaled GPP products in capturing GPP dynamics at independent test sites, or tracking the long-term trajectories GPP before the MODIS-era.

Our analysis revealed that the calibration procedure significantly enhanced the correlation between satellite-derived productivity proxies and site-level GPP at GRA, MF, NF, and SAV sites (Fig. 6). MODIS-based VIs and LCSPP still showed slightly higher correlations with site-level GPP than the calibrated LCREF-based products at some GRA, SAV, and SH sites (Figure S12). The fact that the land cover classes with the greatest improvement in R^2^ after calibration are largely also where some disparities with MODIS remain suggests that the cross-calibration with MODIS has indeed reduced the gap between AVHRR data and site-level photosynthetic activity. Nonetheless, some differences persist and require further attention, particularly in non-forest biomes with relatively spare canopy.

**Fig. 7:**
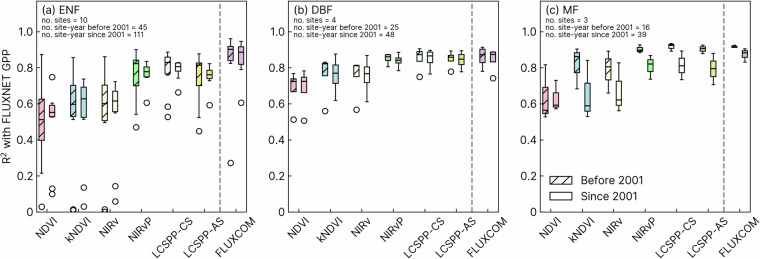
Comparison of the correlation between LCREF-AVHRR derived photosynthesis indices and site-level GPP before and after 2001. For this analysis, 17 sites were selected, each with a minimum of 3 years of observations from both before and after the introduction of MODIS. No significant difference was found in the correlations between the productivity indices and site-level GPP before 2001 compared to after 2001 (one-tailed t-test, *p* > 0.05).

To examine whether applying the calibration derived from the MODIS-era to AVHRR observations before 2001 might lead to a degradation in the reliability of LCSPP, we subsampled 17 FLUXNET (encompassing three land cover classes) sites with at least three years of measurements both before and since 2001, and compared whether there was a systematic difference in R^2^ between the two periods (Fig. 7). We found no significant difference in correlation between the satellite-derived and ground-based productivity observations in the periods before and since 2001. Nevertheless, we caution that the number of available site-year samples during the AVHRR-only period was less than half of the MODIS period, and the differences in R^2^ could also be caused by selection biases or a lack of statistical convergence. An additional comparison with VIs indicated that, even within the AVHRR period, LCSPP has a greater or consistent correlation with GPP than VIs do (paired t-test, one-tailed *p* < 0.05 in all cases). These results suggest that the improvements derived from cross-calibration with MODIS were successfully applied to the AVHRR record before the MODIS era.

## Usage Notes

Overall, we believe the LCREF and LCSPP datasets generated from this study can provide a valuable addition to the data assets for studying long-term vegetation dynamics. We propose that the potential applications of the LCSPP and LCREF datasets include (1) reevaluating the regional greening vs. browning trend under climate change given the improved temporal consistency of the dataset^102^; (2) tracking phenology changes and detecting differences in the seasonality of productivity vs. canopy greenness; (3) constraining global biogeochemical models through data assimilation^19^; (4) establishing a baseline for detecting vegetation stress to be combined with meteorological and observed SIF values during droughts and heatwaves^47^; (5) assessing vegetation resilience after natural or anthropogenic disturbances including fire and deforestation.

Despite the general agreements between the calibrated AVHRR and MODIS-based LCSPP during 2001–2023, we recognized a period with divergent trends in the mid-2000s during which LCSPP-MODIS demonstrated a consistent increase, whereas LCSPP-AVHRR exhibited a slight decline (Fig. 4). While our calibration efforts vastly reduced the magnitude of the differences between the AVHRR and MODIS products compared with the original LTDR AVHRR, we found this difference persisted in the calibrated product, leading to a more subdued increasing trend of the AVHRR-based LCSPP between 2001–2023. We note that applying stringent snow masks, focusing on growing season pixels, and considering non-linear seasonal biases between the reflectance channels could not eliminate the discrepancy. We also believe the solar zenith drift issue to be an insufficient explanation for the difference because the N16, N18, and N19 satellites of this period demonstrated a much smaller range of SZA drifts than the earlier NOAA satellite platforms (see Figure S1).

In a recent effort to generate a spatiotemporally consistent global dataset of GIMMS NDVI (PKU GIMMS NDVI) from 1982–2022, the authors also identified incongruous trends between MODIS and AVHRR during the mid-2000s despite extensive calibration with Landsat^103^. This latest GIMMS NDVI dataset tackled the issue by blending the calibrated GIMMS NDVI product derived from AVHRR between 1982 and 2002 and MODIS NDVI product between 2003 and 2022 to construct the full NDVI time series. Considering the overall superior quality of the MODIS dataset compared with AVHRR and its higher correlation with site-level GPP (Figure S11), we also encourage the users of the LCSPP and LCREF to investigate whether their applications is sensitive to the source of the data stream in the mid-2000s, particularly if analysis of long-term trend is a main concern. Users of the LCSPP and LCREF datasets will also have the option to choose between MODIS or calibrated AVHRR-based reconstruction starting from 2001. Nevertheless, we acknowledge that the incompatible trend issue reveals the limitations of statistical calibration approaches, and further investigation is needed to understand the mechanistic causes of this discrepancy. We also note that the AVHRR and MODIS-based products have minor differences in coverage, and users should make sure a consistent land mask (as in Fig. 4) is used when computing long-term trends.

Compared with traditional VIs that mainly capture changes in vegetation canopy structure, SIF is often considered a more direct proxy of photosynthesis due to its physiological links to the photochemistry. Our results reveal the underappreciated potential of Red and NIR channels for tracking plant photosynthetic dynamics, at least in coniferous forests known to have a strong physiological component of SIF variations. Nevertheless, as the model only uses reflectance datasets as predictors, we caution against directly interpreting the patterns in LCSPP and other SIF-informed photosynthesis proxies datasets as a physiological signal and attributing their variations to physiological stressors. In particular, we do not recommend using LCSPP to retrieve the fluorescence quantum yield Φ_F_ because the machine learning contains no independent constraint for quantum yield. Instead, LCSPP should be considered as a proxy of vegetation activity and especially of chlorophyll absorbed photosynthetically active radiation. The strong predictive capability and data adaptiveness of the machine learning framework allow LCSPP to extract more information about vegetation activity from reflectance channels than VIs computed with a fixed formula such as NDVI, NIRv, and kNDVI, but the trend and variability in the photosynthesis proxy ultimately depend on the quality of underlying reflectance datasets. The current dataset availability limits the spatial resolution of the dataset at 0.05° resolution, which means potential users should be cautious when applying the dataset in ecosystems with high spatial heterogeneities. This intermediate product will be useful for historical vegetation trend analysis while we await dedicated sensors (e.g., FLEX/FLORIS) capable of disentangling the fluorescence emission from reflectance radiance to better quantify the physiological signal in SIF from space^104^.

## Supplementary information

Supplementary Information

## Data Availability

The code used to generate and evaluate the LCSPP data is publicly available at https://github.com/JianingFang/LCSPP_Scientific_Data.
