# Supplementary material for: A long-term reconstruction of a global photosynthesis proxy over 1982–2023
Source: Sci Data. 2025 Mar 3;12:372. doi: 10.1038/s41597-025-04686-6 (PMC11876647; doi:10.1038/s41597-025-04686-6)
Supplement: Supplementary file 1 — Supplementary Information [file 41597_2025_4686_MOESM1_ESM.pdf]

Supplementary Information for “A long-term reconstruction of a global photosynthesis proxy over 1982-2023”

Jianing Fang, Xu Lian, Youngryel Ryu, Sungchan Jeong, Chongya Jiang, Pierre Gentine

Table of Contents:

Figure S1 to S12	Page 2-9
Table S1	Page 10
Table S2	Page 11
Table S3	Page 11
Table S4	Page 12-16
Text S1	Page 16
Text S2	Page 16-17
Text S3	Page 17

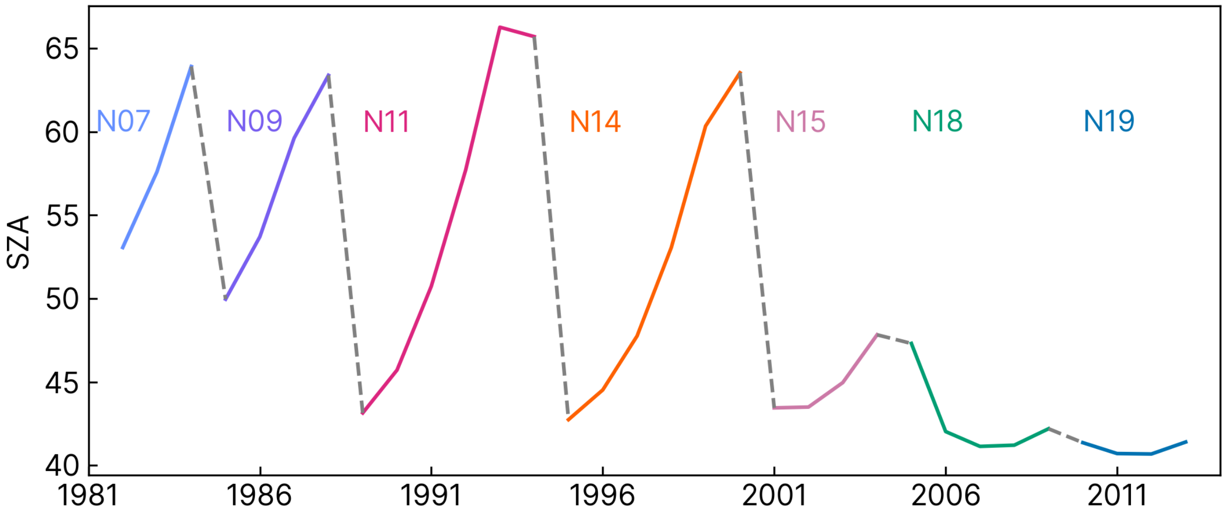

Figure S1: Annual mean solar zenith angle at the time of AVHRR measurements (degrees). Note that 1985, 1988, 2000, 2005, and 2009 contain AVHRR observations from the previous and the next NOAA satellites.

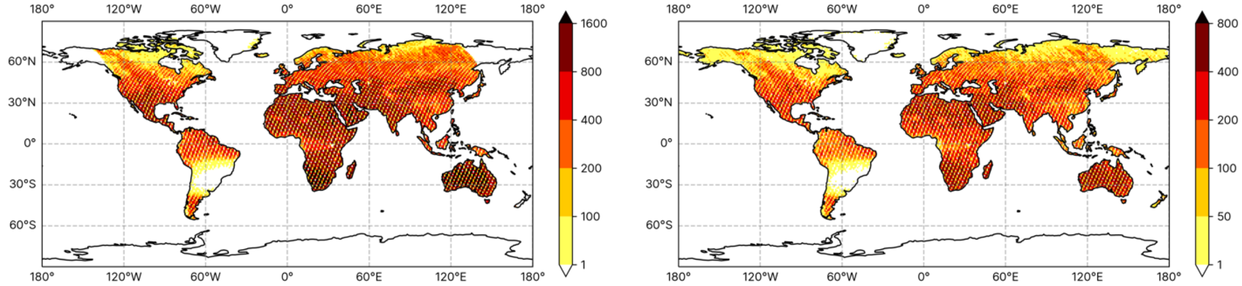

Figure S2: The spatial distributions of training (left) and test samples (right). Individual OCO-2 soundings are collocated with MODIS red and infrared reflectance bands and aggregated to 0.05° CMG to match the resolution of MCD43C1. Cells with fewer than 5 soundings are removed. The number of samples is counted at 1° resolution.

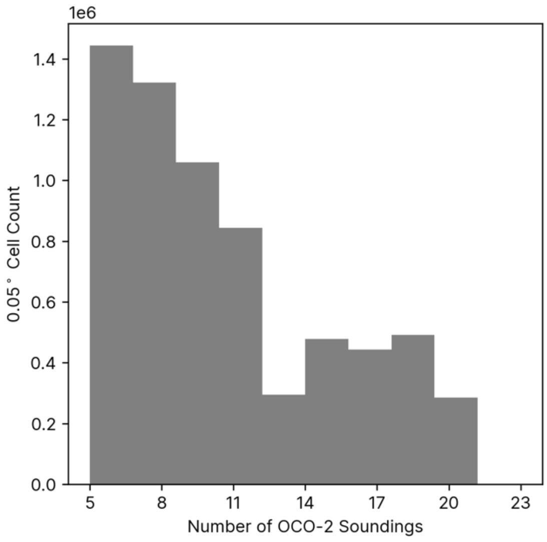

Figure S3: The distribution of OCO-2 sounding counts per 0.05° grid cell within the training dataset.

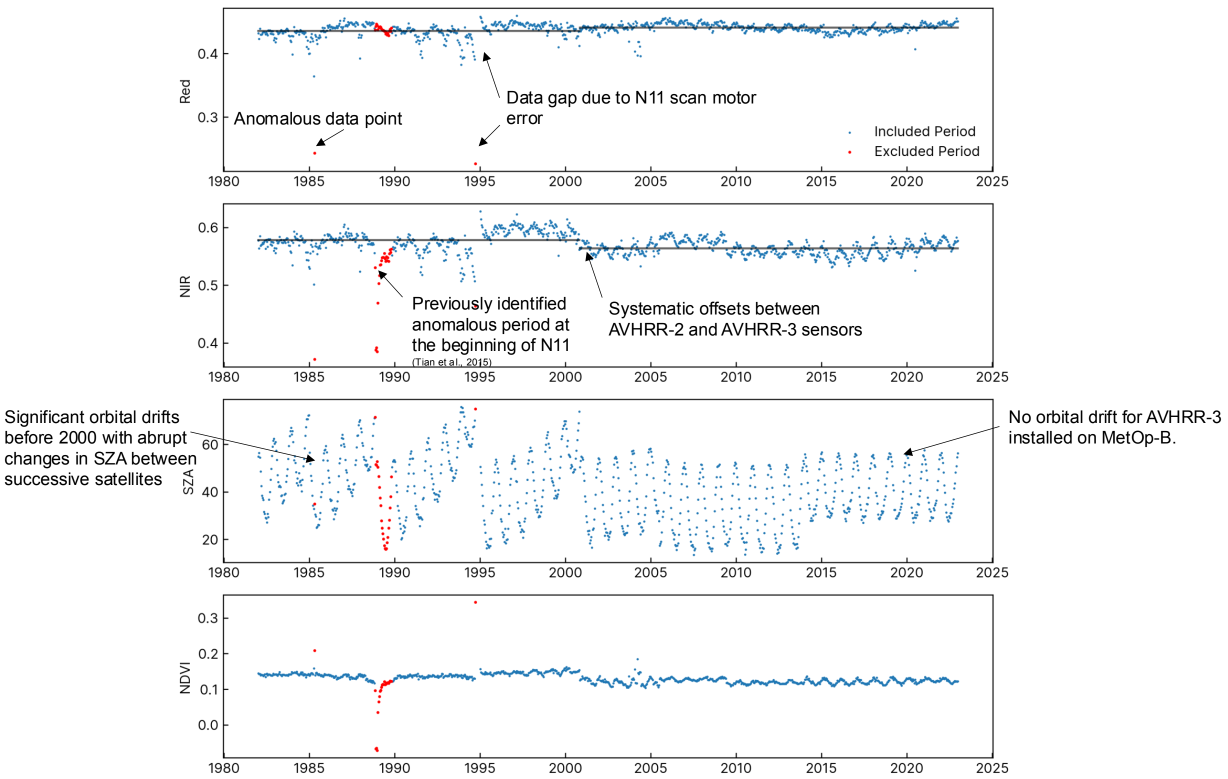

Figure S4. The upper two panels display the red and NIR reflectance of the LTDR AVHRR dataset spatially averaged over 10 calibration PICSs. A 5×5-pixel region centered on the coordinate of each PICSs is used to extract the reflectance. The black horizontal lines indicate the temporal mean between the AVHRR-2 and AVHRR-3 periods. The SZA and NDVI time series averaged across the same set of PICSs are shown in the lower two panels. Data points in red are anomalous periods excluded during the calibration (see Text S1 for justification). We later filled the excluded periods with the seasonal mean of the previous and following years immediately before applying HANTS to gap-fill the complete time series.

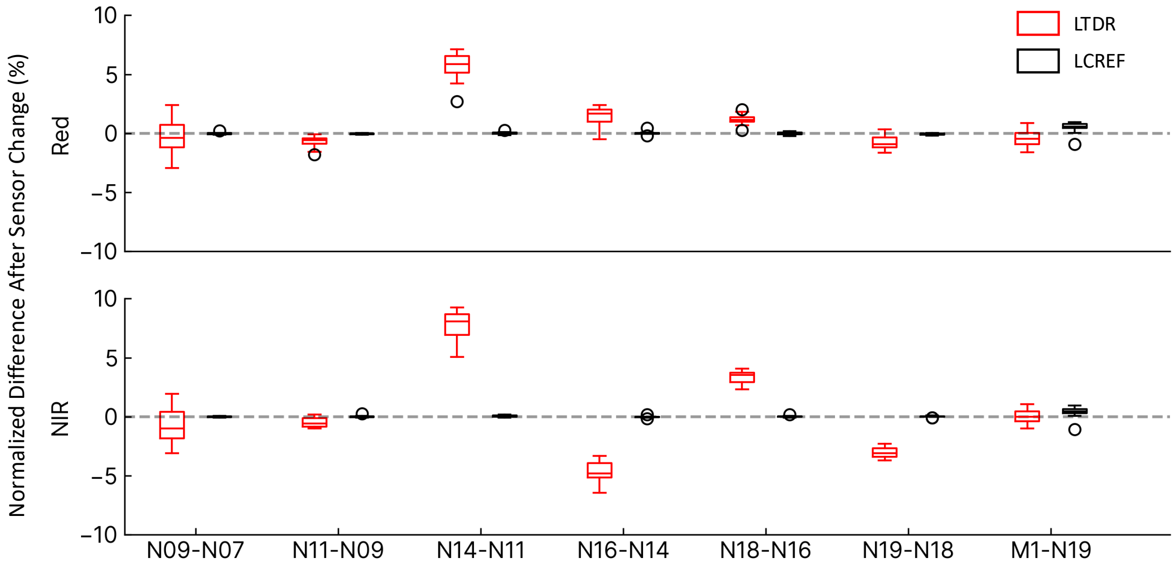

Figure S5. Normalized difference in Red and NIR surface reflectance at 10 validation PICSs after each successive sensor change for both the original LTDR dataset and the calibrated LCREF dataset.

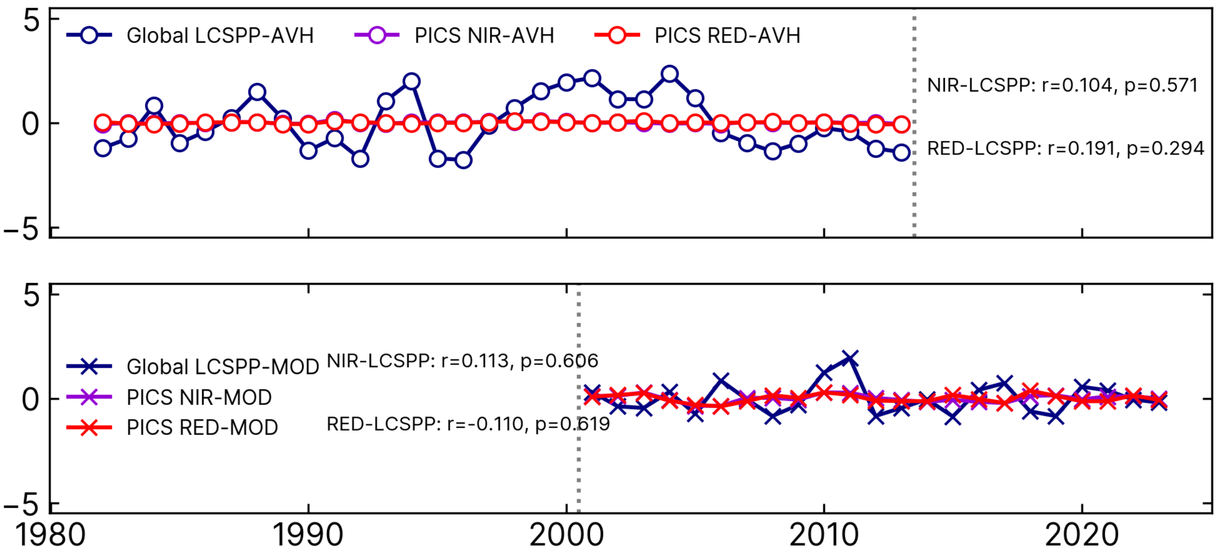

Figure S6: The upper panel shows the median of the annual time series of detrended normalized anomalies (%) of the calibrated red and near-infrared reflectance at the 10 validation PICSs and that of AVHRR-based global growing season mean LCSPP. Values are shown for 1982-2014 when there are expected solar zenith angle drifts within the LTDR dataset. The lower panel shows the median of the detrended normalized anomalies of the MODIS-based reflectance at PICSs and the global annual mean of LCSPP reconstructed from MODIS reflectance.

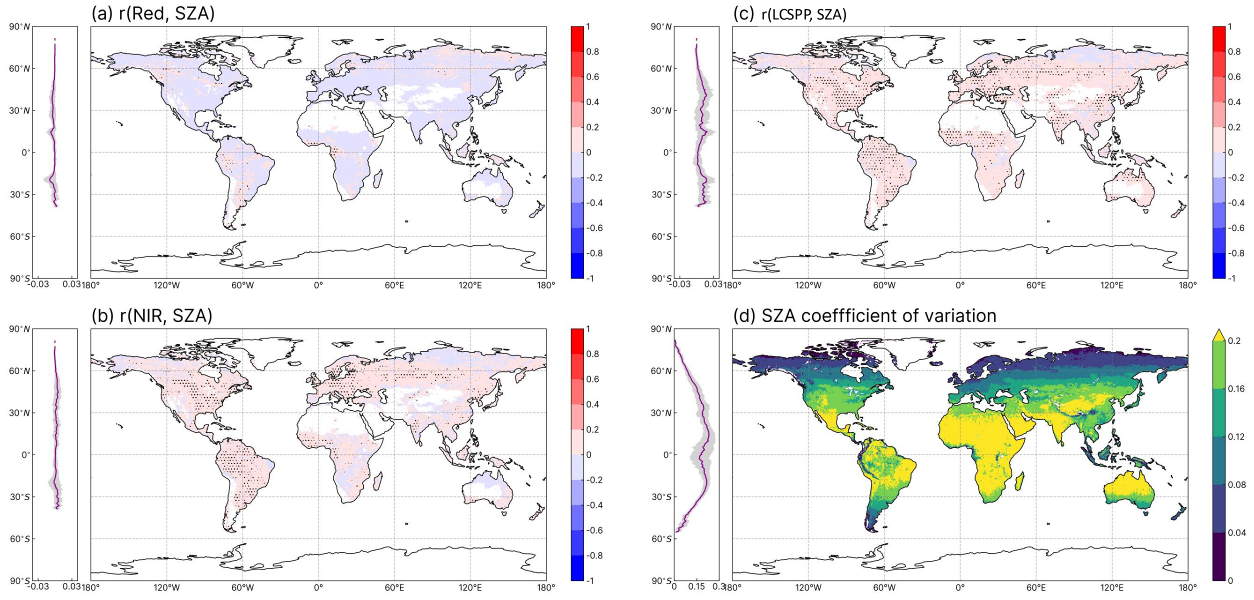

Figure S7. The remaining correlation between the solar zenith angle and the corrected (a) red and (b) NIR channel reflectance and (c) reconstructed LCSPP during the AVHRR period. The shaded area represents regions where the correlation is significant at a $p<0.05$ level. (d) The coefficient of variation (standard deviation/mean) of annual mean SZA at the time of AVHRR measurements.

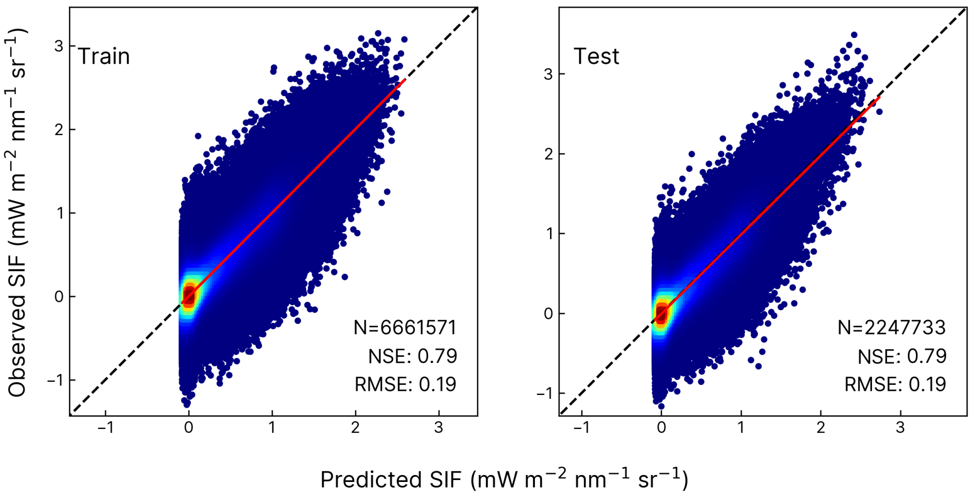

Figure S8. Predicted vs. observed SIF for training and test datasets, for all land cover types combined.

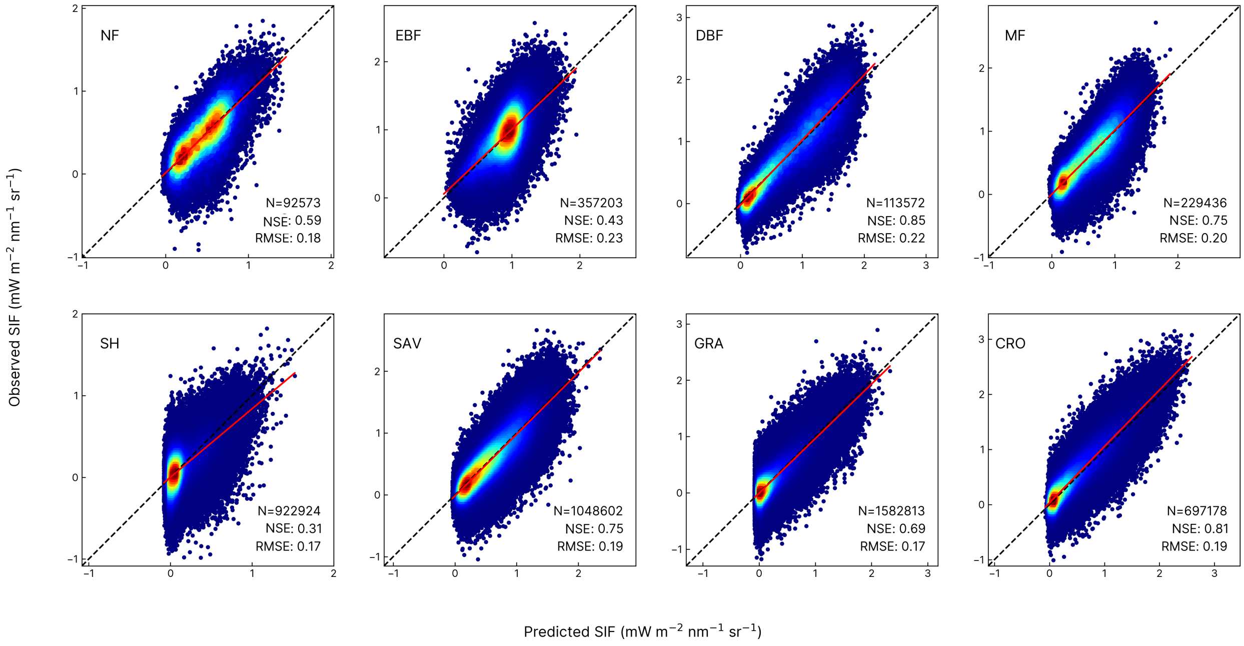

Figure S9. Same as Figure 3 but for the training dataset.

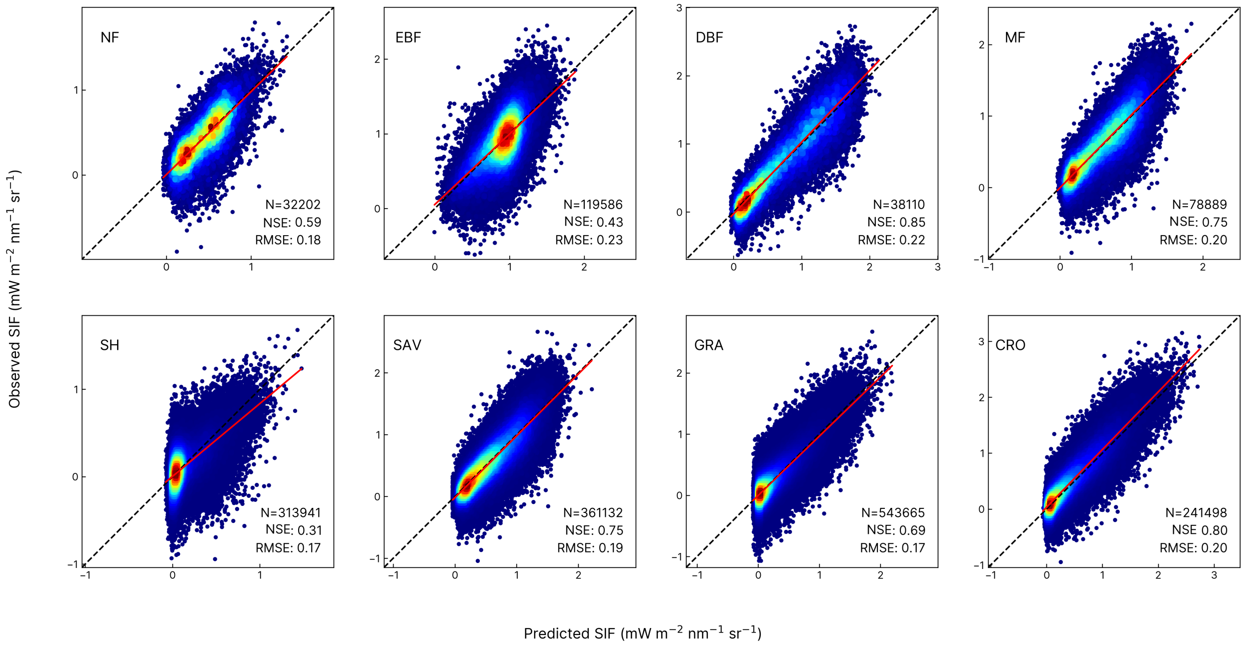

Figure S10. To examine whether the LCSPP model performance is sensitive to how we split the data into training and testing sets, we performed a sensitivity analysis by using data from 2015, 2017, 2018, 2019, 2021, and 2022 for training, and the data from 2016 and 2022 for testing. The resulting model performance is consistent with the original data split.

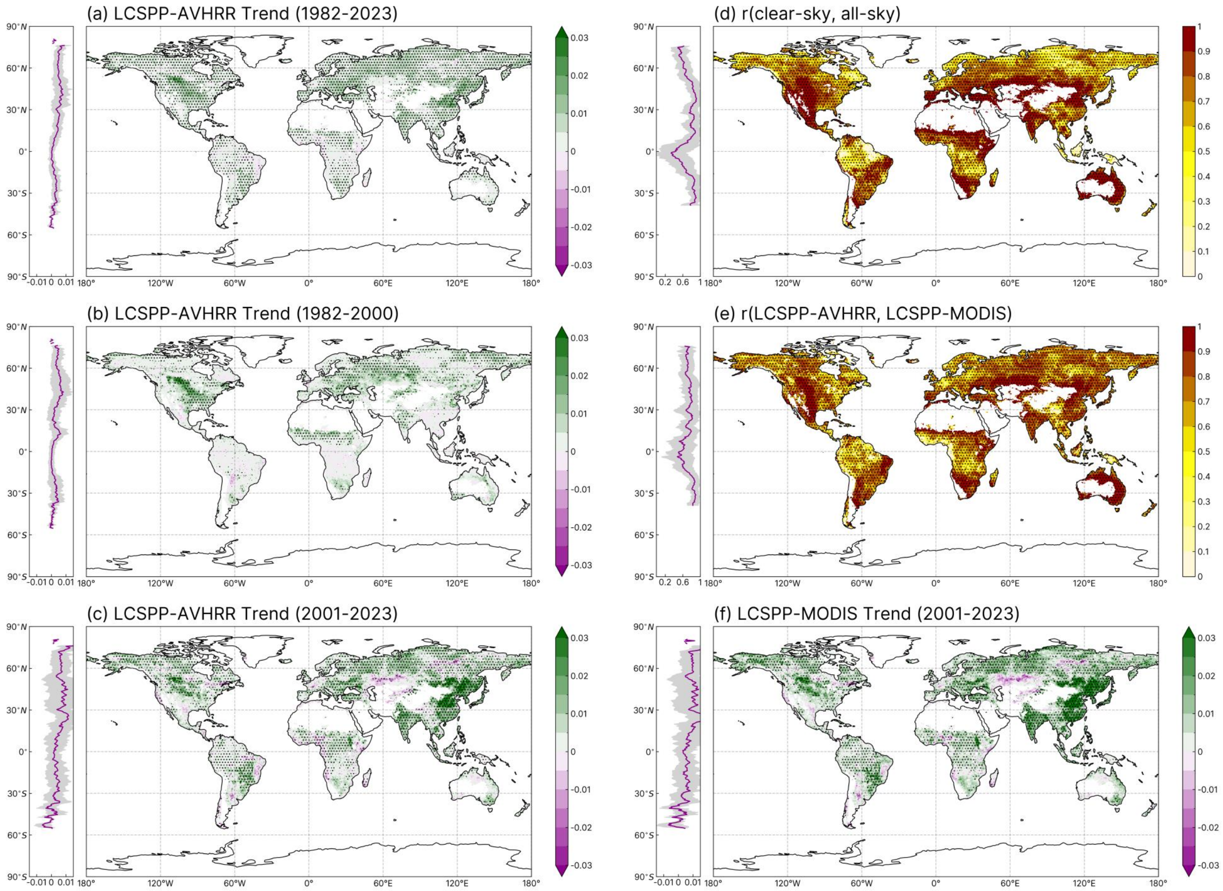

Figure S11: The spatial trends of AVHRR-based and MODIS-based LCSPP, same as Figure 5 in the main text but with trends computed using Theil-Sen slope estimator and significance determined by Hamed and Rao modified Mann-Kendall test with lag 1 autocorrelation and α=0.05.

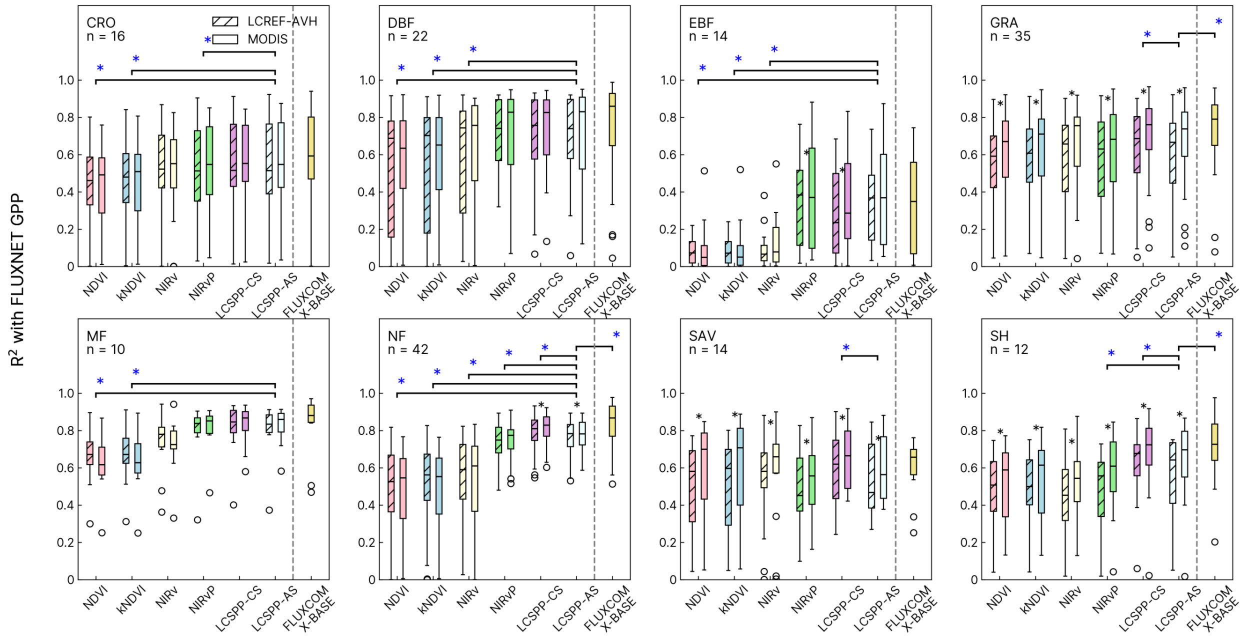

Figure S12: Comparing the strengths of the calibrated AVHRR-based (LCREF-AVH) or MODIS-based VI-GPP and LCSPP-GPP correlations at eddy-covariance sites. We used nighttime partitioned GPP from the FLUXNET 2015 dataset since 2001 for this analysis. The blue asterisk indicates whether the of the correlation with GPP is higher in the case of all-sky LCSPP (LCSPP-AS) than in a reflectance-only VI (paired t-test, one-tailed p<0.05), or whether there is a difference in the correlation with FLUXNET GPP between all-sky LCSPP (LCSPP-AS), FLUXCOM X-BASE, NIRvP, or clear-sky LCSPP (LCSPP-CS) (paired t-test, two-tailed p<0.05). The black asterisk indicates for each type of VI or LCSPP variable, whether the MODIS-based version has a higher correlation with site-level GPP than the AVHRR-based product after calibration (one-tailed p<0.05).

Table S1: A comparison of recent reconstructed SIF-informed Photosynthesis Proxies

Product Name	Reconstruction Algorithm	Predictors	Target SIF Product	Period Available	Temp. & Spatial Resolution
LCSPP (this study)	ANN	MCD43C4: Band 1-2	OCO-2 SIF (757nm)	1982-2021	0.05° biweekly
RSIF[1]	ANN	MYD09A1: Band 1-4	GOME-2 SIF (740nm)	2007-2017	0.5° biweekly
CSIF[2]	ANN	MCD43C4: Band 1-4	OCO-2 SIF (757nm)	2000-2022	0.05° 4day
GOSIF[3]	Cubist regression tree	MCD43C4: EVI MERRA-2: PAR, VPD Ta MCD12C1: Land cover types	OCO-2 SIF (757nm)	2000-2021	0.05° 8day
RTSIF[4]	XGboost	MCD43C4: Band 1-7, CERES PAR MOD11C1: LST MCD12C1: IGBP ISLSCP II: C4 Percentage	TROPOMI SIF (740nm)	2001-2020	0.05° 8day
SDSIF[5]	ANN	MCD43C4: Band 1-4 NDWI ERA5: VPD, Ta	TROPOMI SIF (743-758 nm)	2018-2020	0.05° daily & 4day
LUE-SIF[6]	Semi-empirical LUE model	MCD43C4: NIRv, NDWI LST: MYD11C2	GOME-2 and SCIAMACHY SIF (740nm)	2007-2018	0.05° 8day
HSIF[7]	Random Forest with regionalization constraints + CDF matching harmonization	MCD43C4: Band 1-7	GOME-2 and SCIAMACHY SIF (740nm)	2008-2018	0.05° monthly
ST-LGBM SIF[8]	Spatiotemporal constrained light gradient boosting (ST-LGBM)	MCD43C4: NIRv MERRA-2: PAR, VPD, Ta MCD12C1: LC types OCO-2: Temporal and Spatial Factors	OCO-2 SIF (757nm)	Unknown	0.05° 8day

Table S2: The sensor-origin of LTDR AVHRR data used in this study

AVHRR Platform	Begin Date (YYYYDOY)	End Date (YYYYDOY)
N07	1982001	1985003
N09	1985004	1988312
N11	1988313	1994365
N14	1995001	2000305
N16	2000306	2005181
N18	2005182	2009151
N19	2009152	2013365
M1	2014001	2023181

Table S3. A list of PICSs used for evaluating residual orbital effects in AVHRR

Site	Latitude	Longitude
Arabia2	20.19	51.63
Sudan1*	22.11	28.11
Arabia1*	19.80	47.07
Egypt1	26.61	26.22
Libya3*	23.22	23.23
Libya2	25.08	20.77
Algeria3	30.63	7.83
Mauritania1*	19.51	-8.57
Mali1	19.14	-5.77
Libya4	28.67	23.42
Niger1	20.26	9.64
Algeria1	23.83	-0.76
Mauritania2*	19.78	-8.89
Algeria4*	29.99	5.10
Libya1*	24.65	13.25
Algeria5*	31.16	2.24
Algeria2*	25.99	-0.62
Niger2*	21.33	10.60
Niger3	21.51	7.86
Arabia3	28.80	43.05

* 10 PICSs randomly selected for validation.

Table S4: List of FLUXNET sites used for evaluating LCSPP and VIs

SITE_ID	LAT	LON	IGBP
AR-SLu	-33.4648	-66.4598	MF
AR-Vir	-28.2395	-56.1886	ENF
AT-Neu	47.11667	11.3175	GRA
AU-Ade	-13.0769	131.1178	WSA
AU-ASM	-22.283	133.249	SAV
AU-Cpr	-34.0021	140.5891	SAV
AU-Cum	-33.61518	150.72362	EBF
AU-DaP	-14.0633	131.3181	GRA
AU-DaS	-14.1593	131.3881	SAV
AU-Dry	-15.2588	132.3706	SAV
AU-Emr	-23.8587	148.4746	GRA
AU-Gin	-31.3764	115.7138	WSA
AU-GWW	-30.1913	120.6541	SAV
AU-How	-12.4943	131.1523	WSA
AU-Lox	-34.4704	140.6551	DBF
AU-RDF	-14.5636	132.4776	WSA
AU-Rig	-36.6499	145.5759	GRA
AU-Stp	-17.1507	133.3502	GRA
AU-TTE	-22.287	133.64	GRA
AU-Tum	-35.6566	148.1517	EBF
AU-Wac	-37.4259	145.1878	EBF
AU-Whr	-36.6732	145.0294	EBF
AU-Wom	-37.4222	144.0944	EBF
AU-Ync	-34.9893	146.2907	GRA
BE-Bra*	51.30761	4.51984	MF
BE-Lon	50.55162	4.74623	CRO
BE-Vie*	50.30493	5.99812	MF
BR-Sa1	-2.85667	-54.95889	EBF
BR-Sa3	-3.01803	-54.97144	EBF
CA-Gro	48.2167	-82.1556	MF
CA-Man*	55.87962	-98.48081	ENF
CA-NS1	55.87917	-98.48389	ENF
CA-NS2	55.90583	-98.52472	ENF
CA-NS3	55.91167	-98.38222	ENF
CA-NS4	55.91437	-98.380645	ENF
CA-NS5	55.86306	-98.485	ENF
CA-NS6	55.91667	-98.96444	OSH
CA-NS7	56.63583	-99.94833	OSH

CA-Oas*	53.62889	-106.19779	DBF
CA-Obs*	53.98717	-105.11779	ENF
CA-Qfo	49.6925	-74.34206	ENF
CA-SF1	54.48503	-105.81757	ENF
CA-SF2	54.25392	-105.8775	ENF
CA-SF3	54.09156	-106.00526	OSH
CA-TP1	42.6609361	-80.559519	ENF
CA-TP2	42.7744194	-80.458775	ENF
CA-TP3	42.7068111	-80.348314	ENF
CA-TP4	42.710161	-80.357376	ENF
CA-TPD	42.635328	-80.557731	DBF
CG-Tch	-4.28917	11.65642	SAV
CH-Cha	47.21022	8.41044	GRA
CH-Dav*	46.81533	9.85591	ENF
CH-Fru	47.11583	8.53778	GRA
CH-Lae	47.47833	8.36439	MF
CH-Oe1	47.28583	7.73194	GRA
CH-Oe2	47.28642	7.73375	CRO
CN-Cha	42.4025	128.0958	MF
CN-Cng	44.5934	123.5092	GRA
CN-Dan	30.4978	91.0664	GRA
CN-Din	23.1733	112.5361	EBF
CN-Du2	42.0467	116.2836	GRA
CN-Du3	42.0551	116.2809	GRA
CN-HaM	37.37	101.18	GRA
CN-Qia	26.7414	115.0581	ENF
CN-Sw2	41.7902	111.8971	GRA
CZ-BK1	49.50208	18.53688	ENF
CZ-BK2	49.49443	18.54285	GRA
DE-Geb	51.09973	10.91463	CRO
DE-Hai	51.07921	10.45217	DBF
DE-Kli	50.89306	13.52238	CRO
DE-Lkb	49.09962	13.30467	ENF
DE-Lnf	51.32822	10.3678	DBF
DE-Obe	50.78666	13.72129	ENF
DE-RuR	50.62191	6.30413	GRA
DE-Seh	50.87062	6.44965	CRO
DE-Tha*	50.96256	13.56515	ENF
DK-Eng	55.69053	12.19175	GRA
DK-Sor*	55.48587	11.64464	DBF

ES-Amo	36.83361	-2.25232	OSH
ES-LgS	37.09794	-2.96583	OSH
ES-LJu	36.92659	-2.75212	OSH
FI-Hyy*	61.84741	24.29477	ENF
FI-Jok	60.8986	23.51345	CRO
FI-Let	60.64183	23.95952	ENF
FI-Sod	67.36239	26.63859	ENF
FR-Fon	48.47636	2.7801	DBF
FR-Gri	48.84422	1.95191	CRO
FR-LBr*	44.71711	-0.7693	ENF
FR-Pue	43.7413	3.5957	EBF
GF-Guy	5.27877	-52.92486	EBF
GH-Ank	5.26854	-2.69421	EBF
GL-ZaH	74.47328	-20.5503	GRA
IT-BCi	40.52375	14.95744	CRO
IT-CA1	42.38041	12.02656	DBF
IT-CA2	42.37722	12.02604	CRO
IT-CA3	42.38	12.0222	DBF
IT-Col*	41.84936	13.58814	DBF
IT-Cp2	41.70427	12.35729	EBF
IT-Cpz*	41.70525	12.37611	EBF
IT-Isp	45.81264	8.63358	DBF
IT-La2	45.9542	11.2853	ENF
IT-Lav	45.9562	11.28132	ENF
IT-MBo	46.01468	11.04583	GRA
IT-Noe	40.60618	8.15169	CSH
IT-PT1	45.20087	9.06104	DBF
IT-Ren*	46.58686	11.43369	ENF
IT-Ro1	42.40812	11.93001	DBF
IT-Ro2	42.39026	11.92093	DBF
IT-Tor	45.84444	7.57806	GRA
JP-MBF	44.3869	142.3186	DBF
JP-SMF	35.2617	137.0788	MF
MY-PSO	2.973	102.3062	EBF
NL-Hor	52.24035	5.0713	GRA
NL-Loo*	52.16658	5.74356	ENF
PA-SPn	9.31814	-79.6346	DBF
PA-SPs	9.31378	-79.63143	GRA
RU-Cok	70.82914	147.49428	OSH
RU-Fyo*	56.46153	32.92208	ENF

RU-Ha1	54.72517	90.00215	GRA
SD-Dem	13.2829	30.4783	SAV
SJ-Blv	78.92163	11.83109	SNO
SN-Dhr	15.40278	-15.43222	SAV
US-AR1	36.4267	-99.42	GRA
US-AR2	36.6358	-99.5975	GRA
US-ARb	35.5497	-98.0402	GRA
US-ARc	35.54649	-98.04	GRA
US-ARM	36.6058	-97.4888	CRO
US-Blo*	38.8953	-120.6328	ENF
US-Cop	38.09	-109.39	GRA
US-CRT	41.628495	-83.347086	CRO
US-GBT	41.36579	-106.2397	ENF
US-GLE	41.36653	-106.2399	ENF
US-Goo	34.2547	-89.8735	GRA
US-Ha1*	42.5378	-72.1715	DBF
US-IB2	41.84062	-88.24103	GRA
US-KS2	28.6086	-80.6715	CSH
US-Lin	36.3566	-119.8423	CRO
US-Me1	44.5794	-121.5	ENF
US-Me2	44.4523	-121.5574	ENF
US-Me3	44.3154	-121.6078	ENF
US-Me5	44.43719	-121.56676	ENF
US-Me6	44.3232842	-121.6078	ENF
US-MMS	39.3232	-86.4131	DBF
US-Ne1	41.16506	-96.47664	CRO
US-Ne2	41.16487	-96.4701	CRO
US-Ne3	41.17967	-96.43965	CRO
US-NR1	40.0329	-105.5464	ENF
US-Oho	41.5545	-83.8438	DBF
US-Pfa*	45.9459	-90.2723	MF
US-Prr	65.12367	-147.48756	ENF
US-SRC	31.9083	-110.8395	MF
US-SRG	31.789379	-110.82768	GRA
US-SRM	31.8214	-110.8661	WSA
US-Sta	41.3966	-106.8024	OSH
US-Syv	46.242	-89.3477	MF
US-Ton	38.4316	-120.96598	WSA
US-Tw2	38.1047	-121.6433	CRO
US-Var	38.4133	-120.9507	GRA

US-WCr	45.8059	-90.0799	DBF
US-Whs	31.7438	-110.0522	OSH
US-Wi3	46.634722	-91.098667	DBF
US-Wi4	46.739333	-91.16625	ENF
US-Wi6	46.624889	-91.298222	OSH
US-Wi9	46.618778	-91.081444	ENF
US-Wkg	31.7365	-109.9419	GRA
ZM-Mon	-15.4391	23.2525	DBF

* Sites with at least three years of data in both MODIS and AVHRR periods.

Text S1 Details about anomalous data removal in the AVHRR LTDR record

We excluded three periods from the AVHRR LTDR record during prescreening. We first removed the second half of April, 1985 when we identified a sharp NDVI anomaly over 10 calibration PICSs that we deemed a short-term artifact. The second excluded period (November 1988-October 1989) was a documented low-quality data period at the beginning of the N11[9]. The third period removed (September 16, 1994-December 31, 1994) was due to a scan motor error of the N11 AVHRR sensor on September 13, 1994, causing subsequent data gaps until the end of the N11 mission (https://www.ospo.noaa.gov/Operations/POES/NOAA11/avhrr.html).

Text S2 Additional details for the machine learning model used for AVHRR-MODIS spatial residual correction

As briefly described in Section 2.3 of the main text, we employed both local and global correlational structures between overlapping MODIS and AVHRR observations for calibration. After correcting the biases between AVHRR and MODIS using a local linear model, we calculated the difference between MODIS observations and linearly-corrected AVHRR values as $\gamma_{residual}$, Equation 8 in the main text). We assumed $\gamma_{residual}$ could originate from the non-linear responses of reflectance retrieved by MODIS and AVHRR instruments to environmental variates

such as atmospheric (aerosol and cloud cover) and surface conditions (topography, snow, and soil background noises). To capture the effect of these environmental covariates, we randomly subsampled 3 million samples of the global $\gamma_{residual}$ values from 2001-2023 to form a training dataset (except for 2002, 2010, 2018, which were reserved for validation). The neural network regression predictors are standardized aerosol optical depth, snow depth, cloud cover, elevation, and the linearly corrected AVHRR values. We used a feed-forward neural network with 3 hidden layers and 64 neurons. The model was trained for 10 epochs with a minibatch size 1024 at a learning rate of 0.001. We then added the predicted $\gamma_{residual}$ values to the linearly corrected AVHRR values for the entire AVHRR record.

Text S3: Estimating σ for kNDVI

Kernel NDVI (kNDVI) was developed based on the idea of using a kernel function to represent higher-order interactions between red and near-infrared channels, thereby maximizing the information related to vegetation traits[10]. If a radial-basis function (RBF) is used as the kernel, kNDVI can be expressed as kNDVI = $\tanh\left(\left(\frac{NIR-Red}{2\sigma}\right)^2\right)$, where σ is a hyperparameter to be estimated. As our main application is to derive a nonstationary time series of kNDVI at different eddy-covariance sites, we estimated σ from the mean temporal distance between the NIR and red channels (i.e., $\sigma = \frac{1}{T}\sum_i^N |NIR_i - RED_i|$)[11], which we found in general lead to higher correlation with site-level GPP than estimating σ as the average of the red and NIR for each sample (e.g. kNDVI=tanh $(NDVI^2)$)

References

1. Gentine, P. & Alemohammad, S. H. Reconstructed Solar-Induced Fluorescence: A Machine Learning Vegetation Product Based on MODIS Surface Reflectance to Reproduce GOME-2 Solar-Induced Fluorescence. *Geophysical Research Letters* **45**, 3136–3146 (2018).
2. Zhang, Y., Joiner, J., Hamed Alemohammad, S., Zhou, S. & Gentine, P. A global spatially contiguous solar-induced fluorescence (CSIF) dataset using neural networks. *Biogeosciences* **15**, 5779–5800 (2018).
3. Li, X. & Xiao, J. A Global, 0.05-Degree Product of Solar-Induced Chlorophyll Fluorescence Derived from OCO-2, MODIS, and Reanalysis Data. *Remote Sensing* **11**, 517 (2019).
4. Chen, X. *et al.* A long-term reconstructed TROPOMI solar-induced fluorescence dataset using machine learning algorithms. *Sci Data* **9**, 427 (2022).
5. Hu, J., Jia, J., Ma, Y., Liu, L. & Yu, H. A Reconstructed Global Daily Seamless SIF Product at 0.05 Degree Resolution Based on TROPOMI, MODIS and ERA5 Data. *Remote Sensing* **14**, 1504 (2022).
6. Duveiller, G. *et al.* A spatially downscaled sun-induced fluorescence global product for enhanced monitoring of vegetation productivity. *Earth System Science Data* **12**, 1101–1116 (2020).
7. Wen, J. *et al.* A framework for harmonizing multiple satellite instruments to generate a long-term global high spatial-resolution solar-induced chlorophyll fluorescence (SIF). *Remote Sensing of Environment* **239**, 111644 (2020).
8. Shen, H. *et al.* A Spatiotemporal Constrained Machine Learning Method for OCO-2 Solar-Induced Chlorophyll Fluorescence (SIF) Reconstruction. *IEEE Transactions on Geoscience and Remote Sensing* **60**, 1–17 (2022).

9. Tian, F. *et al.* Evaluating temporal consistency of long-term global NDVI datasets for trend analysis. *Remote Sensing of Environment* **163**, 326–340 (2015).

10. Camps-Valls, G. *et al.* A unified vegetation index for quantifying the terrestrial biosphere. *Sci. Adv.* **7**, eabc7447 (2021).

11. Wang, Q., Moreno-Martínez, Á., Muñoz-Marí, J., Campos-Taberner, M. & Camps-Valls, G. Estimation of vegetation traits with kernel NDVI. *ISPRS Journal of Photogrammetry and Remote Sensing* **195**, 408–417 (2023).
